# Early IKKβ-Dependent Anabolic Signature Governs Vascular Smooth Muscle Cells Fate and Abdominal Aortic Aneurysm Development

**DOI:** 10.3390/cells15030218

**Published:** 2026-01-23

**Authors:** Priscilla Doyon, Ozge Kizilay Mancini, Florence Dô, David Huynh, Gaétan Mayer, Stephanie Lehoux, Huy Ong, Maelle Batardière, Vincent Quoc-Huy Trinh, Ying Wen, Waiho Tang, Sylvie Marleau, Simon-Pierre Gravel, Marc J. Servant

**Affiliations:** 1 Faculty of Pharmacy, Université de Montréal, Montréal, QC H3T 1J4, Canada; priscilla.doyon@umontreal.ca (P.D.); florence.do@umontreal.ca (F.D.); gaetan.mayer@icm-mhi.org (G.M.); sylvie.marleau@umontreal.ca (S.M.); sp.gravel@umontreal.ca (S.-P.G.); 2 The Montreal Heart Institute, Université de Montréal, Montréal, QC H1T 1C8, Canada; 3 Diagnostic and Molecular Pathology, McGill University, Montréal, QC H3A 2M7, Canada; ozge.kizilay@mail.mcgill.ca; 4 Institute for Research in Immunology and Cancer, Université de Montréal, Montréal, QC H3T 1J4, Canada; david.huynh@umontreal.ca (D.H.); maelle.batardiere@umontreal.ca (M.B.); quoc-huy.trinh@umontreal.ca (V.Q.-H.T.); 5 Lady Davis Institute for Medical Research, McGill University, Montréal, QC H3T 1E2, Canada; stephanie.lehoux@mcgill.ca; 6 Institute of Pediatrics, Guangzhou Women and Children’s Medical Centre, Guangzhou Medical University, Guangzhou 510080, China; wenying@gwcmc.org (Y.W.); waiho.tang@gwcmc.org (W.T.)

**Keywords:** abdominal aortic aneurysm, vascular smooth muscle cells, inhibitor of nuclear factor kappa B kinase subunit beta, mammalian target of rapamycin complex 1, anabolic response

## Abstract

Abdominal aortic aneurysm (AAA) is a serious disease with no effective pharmacological therapy. Although inflammation is recognized as a key regulator of AAA, targeting inflammatory pathways once the disease is established does not improve outcomes. Understanding the earliest molecular indicators could clarify precise biological targets and prognostic markers for AAA. Using ApoE-deficient mice, we performed RNA-Seq on suprarenal abdominal aortas (SRAs) from Ang II- and saline-treated mice 24 h after infusion. We further developed a unique model of hyperlipidemic mice in which the expression of the inhibitor of nuclear factor kappa B kinase subunit beta (IKKβ) can be conditionally suppressed in vascular smooth muscle cells (VSMCs). RNA-Seq data revealed early IKKβ-dependent cellular anabolic processes in SRAs, including activation of the mammalian target of rapamycin complex 1 (mTORC1) pathway. Furthermore, deletion of the *Ikbkb* gene in VSMCs significantly reduced the rate of aneurysm rupture in mice exposed to Ang II. In situ analysis further confirmed that the absence of IKKβ in VSMCs is associated with a reduced inflammatory response and the preservation of their contractile phenotypes. Our results reinforce the crucial role of VSMCs in rapid adaptation, leading to deleterious inflammation-dependent remodeling of the vascular wall, and define a previously unrecognized anabolic role of IKKβ in AAA pathogenesis.

## 1. Introduction

Abdominal aortic aneurysm (AAA) is a life-threatening disease characterized by progressive aortic dilation resulting from an imbalance between the synthesis and degradation of extracellular matrix (ECM) components in the vascular wall [1]. Clinically defined as a focal dilatation of the infrarenal aorta (>3 cm), AAA is the most common form of aneurysm in humans. Although increased leukocytic infiltration and a decreased number of vascular smooth muscle cells (VSMCs) in the adventitial and medial layers correlate with the extent of expansive remodeling (i.e., aortic dilatation) and, consequently, with aortic dissections and rupture [2], the precise mechanisms underlying uncontrolled and exaggerated expansive remodeling remain elusive. Therefore, there is no effective pharmacological therapy to stabilize or reverse the progression of small AAA, and open or endovascular surgery remains the only option for treating this debilitating disease [3].

Risk factors for AAA development are well documented and include age, male sex, smoking, hypertension, low levels of high-density lipoprotein (HDL) cholesterol, and a family history of AAA [1]. Inflammation is implicated in many risk factors for AAA development, and most studies focus on inflammatory cells such as macrophages, neutrophils, CD4^+^ T cells, and mast cells in ECM remodeling [4,5]. Another feature common to numerous risk factors for AAA development is the presence of various anabolic processes [6]; yet this aspect is not studied as extensively as inflammation.

VSMCs that reside in the tunica media are the major component of the aortic wall. They contribute to vascular hemostasis by secreting cross-linked collagen and elastin. Under normal conditions, VSMCs are quiescent and non-migratory, displaying a contractile differentiated phenotype [7,8]. However, VSMCs are not terminally differentiated and undergo reversible changes (or dedifferentiation) towards a so-called “synthetic” phenotype, a process referred to as phenotypic modulation, cell plasticity, or phenotypic switch, in response to vascular injury. VSMCs phenotypic switch is characterized by reduced expression of SMC-selective differentiation markers (smooth muscle protein 22-α (SM22A), α-smooth muscle actin (ACTA2)) and increased VSMCs growth (hypertrophy and/or hyperplasia), migration, senescence, ECM metabolism (increased matrix metalloproteinases (MMPs) expression), reactive oxygen species (ROS) production, and inflammatory responses [9]. Interestingly, this “inflammatory/hyperactive-synthetic state” of VSMCs was first observed and characterized in human and animal models following angioplasty, in hypertension-associated remodeling events in small arteries, and in atherosclerosis [8,10]. Importantly, it has recently been described in humans and mice with AAA [11,12,13]. However, the role of VSMCs-dependent anabolic pathways in AAA development and subsequent rupture and dissection remains unclear.

We previously demonstrated that the inhibitor of nuclear factor kappa B kinase subunit beta (IKKβ) is expressed in VSMCs and plays key roles in inflammatory signaling events in response to vasoactive peptides and pathogens [14,15,16,17]. By controlling the activation of nuclear factor kappa B (NF-κB) transcription factors [18] and stabilizing mRNAs encoding numerous proinflammatory cytokines [19], IKKβ is a major effector of the innate and adaptive immune systems. It is therefore considered a druggable anti-inflammatory target [20]. Although previous mouse models of AAA have shown that anti-inflammatory strategies can effectively attenuate aneurysm formation [21,22,23], quenching vascular inflammation using classical pharmacological approaches (e.g., indomethacin) or inhibiting the immune system (e.g., mast cell inhibition, anti-interleukin-1β therapy) does not influence the progression of AAA in humans [24]. Thus far, the apparent failure to identify a druggable target for AAA could be explained by the inability of anti-inflammatory approaches to target the detrimental synthetic state of VSMCs that occurs during AAA development [12] or by a lack of a complete picture of the molecular events that drive this aortopathy.

In this study, we used a murine model of AAA comprising Angiotensin II (Ang II)-infused mice on a hyperlipidemic apolipoprotein E^−/−^ (*ApoE^−/−^*) genetic background to demonstrate the rapid and unique anabolic molecular signature orchestrated by IKKβ. Furthermore, preventing IKKβ expression specifically in VSMCs reduced the occurrence of the synthetic phenotype and attenuated AAA formation. Our data suggest that controlling the anabolic response through IKKβ could be exploited to treat AAA.

## 2. Methods

Full methods are provided in the Supplementary Materials.

### 2.1. Animal Experiments

All animal experiments were conducted in a specific pathogen-free barrier facility in accordance with institutional guidelines and local protocols (18-097 and 18-098) approved by the Ethics Committee of the Université de Montréal and in accordance with the European Communities Council Directive 2010/63/EU for the protection of animals used for experimental purposes. We employed the Ang II-infused apolipoprotein E^−/−^ (*ApoE*^−/−^) C57BL/6J mouse model. For osmotic pump implantation, mice were anesthetized with a 3% isoflurane/oxygen (2 L/min, 100% O_2_) gas mixture. One hour prior, mice received a subcutaneous injection of 10 mg/kg carprofen (50 mg/mL), diluted 1:100 with saline, then further diluted with 37 °C sterile saline to a total volume of 1 mL to maintain hydration and circulating volume. Mice were placed under an anesthesia mask on a pre-heated platform, with isoflurane reduced to 2% and oxygen to 1 L/min to avoid deep anesthesia. Anesthesia depth was confirmed by the absence of palpebral reflex or withdrawal response, and osmotic pumps were implanted. For the following two days, mice received postoperative analgesia with carprofen (10 mg/kg) once daily (SID). For organ harvesting, animals were sacrificed 1, 2, 4, 8 or 28 days post-minipump implantation. Mice were euthanized by exsanguination under 3% isoflurane/oxygen (2 L/min, 100% O_2_) anesthesia.

### 2.2. Statistics

Data are presented as mean ± SEM. Fisher’s exact test and the log-rank (Mantel–Cox) test were used to assess statistical significance of aneurysm incidence and mouse survival, respectively. An unpaired two-tailed Student’s *t*-test was used to compare two groups, and a two-way ANOVA with Tukey’s multiple-comparison test was used to evaluate two independent variables. Statistical analyses were performed using Prism 9.0. *p* < 0.05 was considered statistically significant.

## 3. Results

### 3.1. Early Anabolic Response Defines the Molecular Signature of Aortopathy in the Ang II-Treated ApoE-Deficient Mouse-AAA Model

**Figure 1:**
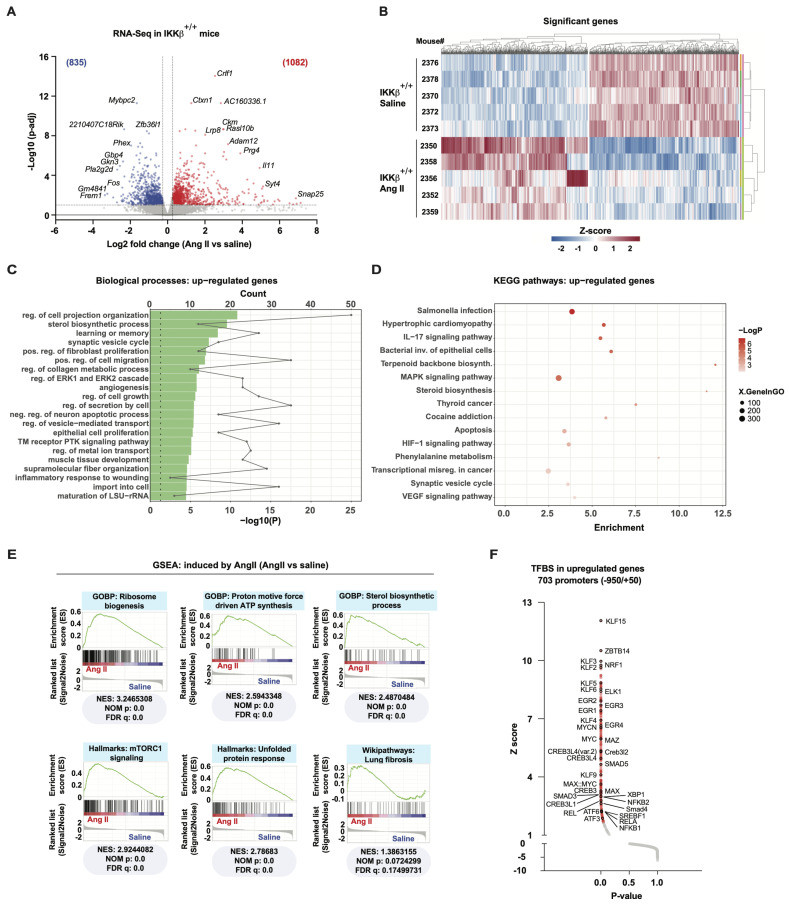
Early anabolic response defines the molecular signature of aortopathy in the Ang II-infused *ApoE*-deficient mouse-AAA model. (**A**) Volcano plot of differentially expressed transcripts (DET) in SRAs after 24 h of Ang II treatment. Each point shows the average fold change and associated *p*-adjusted value for a single transcript, comparing Ang II (*n* = 5 mice) with saline (*n* = 5 mice). Colored numbers indicate the number of significantly induced (red) and reduced (blue) transcripts at the given threshold (dotted lines), as indicated in Materials and methods. (**B**) Heatmap of significant DET. Expression fold changes have been transformed to z-scores, and clustering has been performed on rows (mice) and columns (transcripts). (**C**) Functional classification of significant upregulated DET with Metascape for the Gene Ontology: Biological Processes collection of gene sets. Green bars indicate *p*-values (−Log10), and dots indicate the number of genes per pathway. (**D**) Functional classification as in C, but for KEGG pathways, using an enrichment bubble plot. The X-axis represents the enrichment factor (number of genes/all genes from pathway), XgeneInGO indicates the number of genes (bubble size), and −LogP indicates significance based on the *p*-value. (**E**) Gene set enrichment analyses (GSEAs) on all normalized transcripts in control (saline) and Ang II-treated mice. NES: Normalized enrichment score; NOM p: nominal *p*-value; FDR q: false discovery rate-adjusted q-value. (**F**) Over-representation of TFBS in the promoter (−950 to +50) of significantly induced DET in Ang II-treated versus saline-treated mice. Red dots indicate significant over-representation with a *p*-value < 0.05. Labeled dots also have a black outline.

Tissue remodeling leading to aortic dissection occurs rapidly in 8–10-week-old male *ApoE*-deficient mice fed a standard diet and infused with Ang II [25,26]. Most fatalities from aortic rupture occur within the first seven days of Ang II infusion and correlate with phagocytic markers in AAA lesions, concomitant with elastin fiber disruption [27]. Therefore, most studies focusing on the molecular etiology of AAA employ whole aortas or suprarenal abdominal aortas (SRAs) isolated from animals exposed to Ang II for 2 to 7 days [28]. In these settings, vascular wall remodeling (primarily ECM metabolism) and widespread increases in inflammatory genes are already present, which could mislead and complicate the interpretation of early cellular, molecular, and biochemical responses underlying this aortopathy. We therefore conducted bulk RNA-Seq analysis on SRAs from both Ang II-treated and saline-infused IKKβ^+/+^ mice, specifically examining responses 24 h after infusion of Ang II at 1000 ng/kg/min to capture the initial phases of the aortic stress response. A volcano plot of the 1917 differentially expressed genes (DEGs) revealed distinct patterns in aortic gene expression between Ang II-treated and saline-infused IKKβ^+/+^ mice (Figure 1A,B). Next, we performed functional analyses of these differentially expressed transcripts (DET) to characterize the transcriptomic signature associated with early Ang II signaling in SRAs. First, functional analysis of significantly induced DET using the Gene Ontology Biological Process (GOBP) collection of gene sets revealed enrichment of pathways associated with cell growth, cell proliferation, extracellular matrix remodeling, angiogenesis, and sterol biosynthesis (Figure 1C). A similar analysis using the KEGG pathway collection of gene sets identified enrichment of pathways associated with hypertrophic disease, pro-mitogenic and inflammatory signaling, as well as terpenoid and steroid biosynthesis, all linked to cholesterol biosynthesis (Figure 1D). Strikingly, these two functional analyses indicate that early Ang II signaling in SRAs leads to transcriptomic changes associated with long-term and chronic hypertrophic disease. To better delineate this early transcriptomic signature, we performed Gene Set Enrichment Analyses (GSEAs). This computational and statistical method does not apply cutoffs and considers all genes within a gene set [29]. GSEAs confirmed the enrichment of the sterol biosynthesis gene set (Figure 1E). Surprisingly, of the 18 enzymes involved in cholesterol biosynthesis, 15 had a fold change > 1.2, and 11 were significantly upregulated (adjusted *p*-value < 0.1) (Supplementary Figure S1A). In addition to cholesterol biosynthesis, GSEAs revealed enrichment of metabolic pathways that functional analyses could not detect, such as ribosome biosynthesis, mitochondrial ATP synthesis, and mammalian target of rapamycin complex 1 (mTORC1) signaling, a major regulator of metabolism and vascular muscle growth (Figure 1E). GSEAs also revealed enrichment for the unfolded protein response (UPR) and fibrosis, suggesting extensive protein synthesis and extracellular matrix remodeling that could quickly ignite stress and pro-inflammatory responses. To help identify transcription factors that could mediate these transcriptomic changes in response to Ang II in SRAs, we examined the over-representation of transcription factor binding sites (TFBS) within the promoters of significantly induced DET. These analyses showed enrichment of TFBS for transcription factors linked to vascular remodeling, anabolic and fibrotic events, including suppressor mothers against decapentaplegic (SMADs), cAMP-responsive element-binding (CREB), ETS transcription factor ELK1 (ELK1), early growth response (EGR), MYC proto-oncogene bHLH transcription factor (MYC), X-box binding protein 1 (XBP1), activating transcription factors (ATFs), NF-κB and Krüppel-like factors (KLFs) (Figure 1F). These findings support early anabolic events in the initiation of AAA, mainly composed of cellular processes that were recently shown to be directly controlled by the mTORC1 pathway [6,30,31].

### 3.2. IKKβ Regulates VSMCs Phenotypic Switch from Contractile to Synthetic State in AAA Development

**Figure 2:**
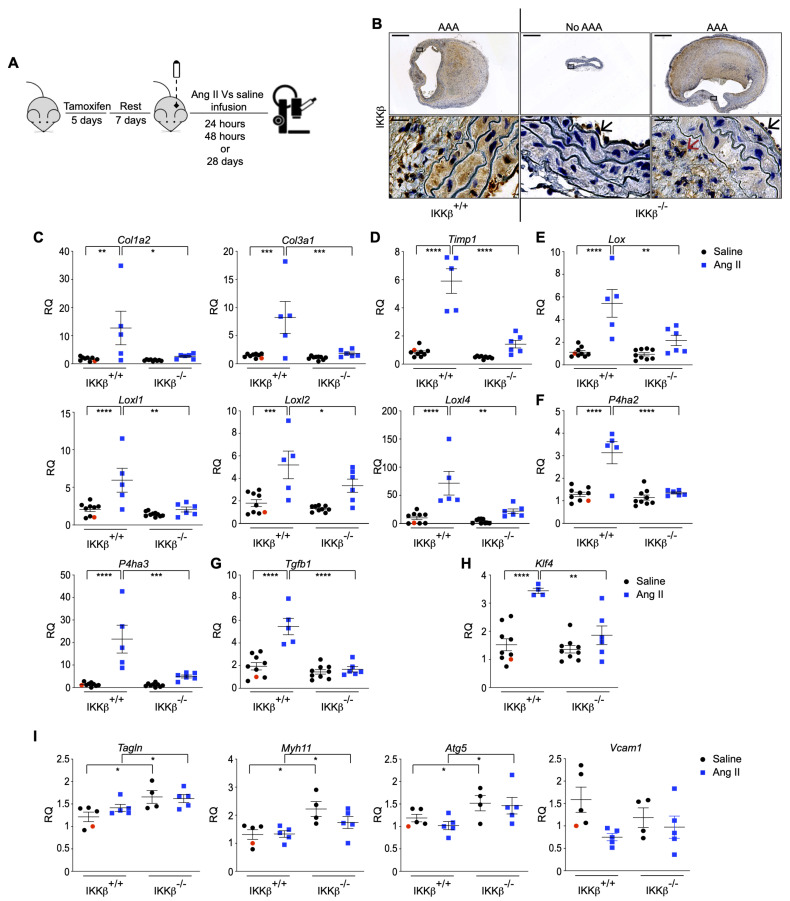
IKKβ is required for VSMCs phenotypic switching. (**A**) Schematic of the experimental procedure. Eight-week-old SMA-CreER^T2^ IKKβ^Flox/Flox^ *ApoE*^−/−^ (IKKβ^−/−^) and their counterpart SMA-CreER^T2^ IKKβ^WT/WT^ *ApoE*^−/−^ (IKKβ^+/+^) male mice were injected with tamoxifen for 5 consecutive days. After 7 days of rest, mice were infused with saline or Ang II at 1000 ng/kg/min for 24 h, 48 h, or 28 days. (**B**) Representative immunohistochemistry of conditional IKKβ deletion in VSMCs of SRAs from IKKβ^+/+^ (*n* = 2) and IKKβ^−/−^ (*n* = 4) mice infused with Ang II for 28 days. Black and red arrows indicate intimal and adventitial staining, respectively, demonstrating specific IKKβ knockout in the media. Scale bars: 600 µm; magnification scale bar: 37.5 µm. (**C**–**I**) qPCR analysis of various mRNA expression in aorta lysates from IKKβ^+/+^ and IKKβ^−/−^ mice infused with saline, or IKKβ^+/+^ and IKKβ^−/−^ mice infused with Ang II (1000 ng/kg/min) for 28 days (**C**–**H**) or for 24 h (**I**). Data are shown as mean ± SEM. * *p* < 0.05, ** *p* < 0.01, *** *p* < 0.001, **** *p* < 0.0001, 2-way ANOVA with Tukey’s multiple-comparison test. RQ: Relative quantification. Red circles represent the reference. (**C**) Relative expression of *Col1a2*, *Col3a1*; (**D**) Relative expression of *Timp1*; (**E**) Relative expression of *Lox*, *Loxl1*, *Loxl2*, *Loxl4*; (**F**) Relative expression of *P4ha2*, *P4ha3*; (**G**) Relative expression of *Tgfb1*. Saline: IKKβ^+/+^ (*n* = 9) and IKKβ^−/−^ (*n* = 9); Ang II: IKKβ^+/+^ (*n* = 5) and IKKβ^−/−^ (*n* = 6); (**H**) Relative expression of *Klf4*. Saline: IKKβ^+/+^ (*n* = 9) and IKKβ^−/−^ (*n* = 9); Ang II: IKKβ^+/+^ (*n* = 4) and IKKβ^−/−^ (*n* = 6); (**I**) Relative expression of *Tagln*, *Myh11*, *ATG5*, and *Vcam1*. Saline: IKKβ^+/+^ (*n* = 5) and IKKβ^−/−^ (*n* = 4); Ang II IKKβ^+/+^ (*n* = 5) and IKKβ^−/−^ (*n* = 5).

The inflammatory effector kinase IKKβ, which is absolutely required for activation of the canonical NF-κB transcription factor pathway, was previously shown by our group to regulate mTORC1 activation in response to Ang II, thereby controlling both the inflammatory and anabolic-hypertrophic effects of the vasoactive peptide [16]. Given the well-recognized inflammatory nature of AAA and the observed anabolic mTORC1 signature above, we next examined the role of IKKβ in the development of experimental AAA by generating hyperlipidemic mice in which the *Ikbkb* gene is specifically inactivated in VSMCs. We refer to this unique transgenic homozygous hypercholesterolemic mouse model as SMA-CreER^T2^Ikkβ^Flox/Flox^*ApoE*^−/−^, in which IKKβ expression is temporally (tamoxifen injection) and selectively (CreER^T2^ under the control of the *Acta2* promoter; refer to Supplementary Figure S1B) prevented in *ApoE*^−/−^ VSMCs on a C57BL6 background. At 8 weeks of age, male SMA-CreER^T2^Ikkβ^Flox/Flox^*ApoE*^−/−^ (IKKβ^−/−^) and their control littermates SMA-CreER^T2^*ApoE*^−/−^ (IKKβ^+/+^) mice were subjected to 5 consecutive days of tamoxifen injection. After an additional 7 days, mice were infused using the classical AAA-induction protocol, in which animals were exposed to either saline or Ang II for 28 days, a time point at which vessel wall expansion, aneurysm formation, and ruptures can all be assessed simultaneously (Figure 2A). Tamoxifen infusion efficiently induced cell-specific conditional deletion of IKKβ in VSMCs, as no kinase staining was detected in the media compared with the intima and adventitia (Figure 2B). Morphologically, the aortas of saline-infused IKKβ^−/−^ animals did not differ from those of saline-infused control IKKβ^+/+^ mice (Supplementary Figure S1C). Moreover, IKKβ deficiency did not affect body weight, end-point systolic blood pressure, plasma cholesterol, or triglycerides. (Supplementary Figure S1D,E).

The failure of lipid-lowering, anti-hypertensive, anti-inflammatory and anti-protease therapies (fenofibrate, statins, β-blockers, ACE inhibitors, IL-1β neutralization, mast cell inhibition, indomethacin, doxycycline) to reduce AAA progression in humans [24] suggests the involvement of yet undiscovered or underestimated essential elements, such as VSMC phenotypic switch, that likely remain unresponsive to these therapies. In experimental models, Ang II triggers a VSMCs phenotypic switch, in part through mTORC1-dependent hypertrophic and anti-autophagic effects, cellular responses recently documented to lead to early aortopathy in *ApoE*-deficient mice [32,33]. As we previously showed the role of IKKβ in the mTORC1-dependent hypertrophic phenotypic response of VSMCs [16], we next verified the expression of synthetic and contractile markers in aortic tissues derived from IKKβ^+/+^ and IKKβ^−/−^ animals treated with Ang II. Aortas isolated from animals infused with Ang II for 28 days showed induced expression of VSMCs synthetic and fibrotic marker transcripts involved in collagen expression and remodeling (maturation and turnover), including the collagen type I alpha 2 chain (*Col1a2*) and the collagen type III alpha 1 chain (*Col3a1*), the prolyl-4-hydroxylase 2 (*P4ha2*) and 3 (*P4ha3*), the lysyl oxidase (*Lox*), the lox like 1 (*Loxl1*), 2 (*Loxl2*) and 4 (*Loxl4*) (Figure 2C,E,F). This was also accompanied by the homeostatic induction of the protective effectors including the tissue inhibitor of metalloproteinase 1 (*Timp1*) and the cytokine transforming growth factor beta 1 (*Tgfb1*) [34] (Figure 2D,G). The ablation of IKKβ in the vascular wall completely blunted their expression (Figure 2C–G). Remarkably, the expression of the key initiating transcription factor governing this phenotypic switch, *Klf4*, was significantly reduced in IKKβ^−/−^ littermates exposed to Ang II (Figure 2H). Furthermore, protein extracts from the SRAs of animals treated with Ang II for 28 days strongly support the preservation of the contractile phenotype in IKKβ^−/−^ animals, with a significant decrease in the phosphorylation of p65 (NF-κB subunit) and MMP9 expression, alongside an increase in the expression of the master regulator of SMC plasticity TET2 [35], and αSMA (Supplementary Figure S2A–C). Together, our data suggest the absence of IKKβ in VSMCs contributes to the preservation of the contractile phenotype, thereby reducing the need of a protective response in the presence of Ang II.

Using SRAs isolated from animals treated with Ang II for 24 h, we further observed that preservation of a contractile phenotype is a very early event in our IKKβ-deficient model. The relative expression of classical contractile markers myosin heavy chain 11 (*Myh11*) and transgelin (*Tagln*), as well as the autophagic marker autophagy related 5 (*Atg5*) [33], was significantly increased after a 24 h Ang II infusion, a condition in which the inflammatory marker *Vcam1* was not yet significantly modulated (Figure 2I). In summary, our findings underscore the crucial role of IKKβ in mediating the phenotypic switch of VSMCs from a contractile to a synthetic state. The absence of IKKβ in the vascular wall preserves the contractile phenotype and suppresses the expression of synthetic markers associated with aortic remodeling and aneurysm formation.

### 3.3. Early IKKβ-Dependent Transition of VSMCs into Phagocytic-like Cells and Anabolic Signature in AAA Development

**Figure 3:**
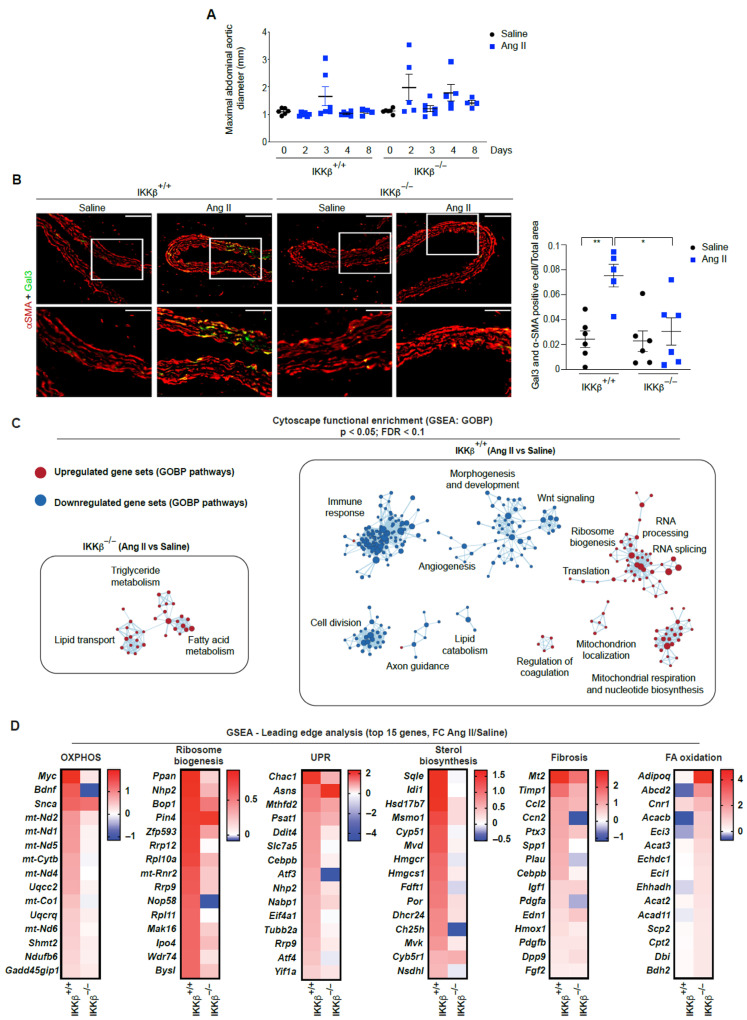
IKKβ drives early transition of VSMCs toward a degradative phenotype coupled to an anabolic response. (**A**) Maximal abdominal aortic diameter in mice from both groups infused with saline or Ang II at 1000 ng/kg/min at different endpoints. Data were quantified and presented as mean ± SEM. (**B**) Representative α-SMA and Gal3 immunofluorescent co-staining of SRAs from IKKβ^+/+^ (*n* = 6) and IKKβ^−/−^ (*n* = 6) mice infused with saline at day 0, and from IKKβ^+/+^ (*n* = 5) and IKKβ^−/−^ (*n* = 6) mice infused with Ang II at 1000 ng/kg/min for 4 days, with quantification. Scale bars: 40 µm; magnification scale bars: 20 μm. Data were quantified and presented as mean ± SEM. * *p* < 0.05, ** *p* < 0.01, 2-way ANOVA with Tukey’s multiple-comparison test. (**C**) Cytoscape (Version 3.10.1) network representation of enriched gene sets in IKKβ^+/+^ and IKKβ^−/−^ mice in response to early Ang II signaling. Nodes represent gene sets; node size indicates the number of genes within each set; and edges indicate similarity between gene sets. Red indicates upregulated gene sets, and blue indicates downregulated gene sets. (**D**) Heatmap representations of transcript fold change (Log2) in response to Ang II treatment (versus saline) for selected GSEAs pathways. Transcripts are ranked by fold change, from highest (red) to lowest (blue).

VSMC dedifferentiate and initiate expression of macrophage markers upon cholesterol exposure [36]. Given the importance of the early sterol biogenesis signature (Figure 1E and Supplementary Figure S1A) and the roles of mTORC1 and KLF4 in the rapid polarization of VSMCs towards phagocytic-like cells in aortic diseases, including dissections and ruptures [11,37,38,39], we next examined the contribution of IKKβ to the development of early aortic lesions and the presence of a degradative VSMCs phenotype. As shown in Figure 3A, from day 2 to day 8 of Ang II infusion, no significant aortic enlargement was observed. However, the absence of IKKβ in the vascular wall reduced the occurrence and severity of aneurysms and ruptures (Table 1), effects that correlated with a reduction in VSMCs expressing the macrophage marker galectin 3 (Gal3) (Figure 3B). We thus hypothesized that IKKβ was responsible for the mTORC1 transcriptomic signature observed in wild-type mice in response to Ang II (Figure 1E). To decipher the role of IKKβ in this response, we first performed GSEAs in IKKβ^+/+^ and IKKβ^−/−^ mice infused with Ang II for 24 h and generated networks of enriched gene sets. Strikingly, these analyses showed that anabolic processes linked to ribosome biogenesis, mRNA translation and mitochondrial respiration are not induced in IKKβ^−/−^ mice in response to Ang II (Figure 3C). However, knockout animals still respond to Ang II, as shown by volcano plots and a heatmap representation of DET (Supplementary Figure S3A,B), and by the induction of genes linked to lipid catabolism (Figure 3C,D). This suggests that IKKβ has a profound role in determining the metabolic fate of vascular cells in response to Ang II. Further analyses of the most induced transcripts confirm that the individual transcripts associated with oxidative phosphorylation (OXPHOS), ribosome biogenesis, UPR, sterol biosynthesis and fibrosis are not induced or are less induced in IKKβ^−/−^ mice in response to early Ang II signaling, and that the lipid catabolism signature is specific to IKKβ^−/−^ mice (Figure 3D). Importantly, the anabolic signature of IKKβ^+/+^ mice is strongly associated with mTORC1, a main regulator of these cellular processes [6,30,31].

**Figure 4:**
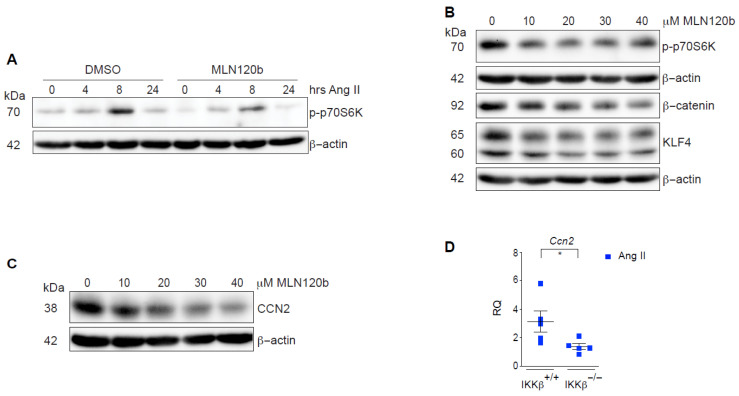
Fate-shaping properties of IKKβ in cultured VSMCs. (**A**) Quiescent VSMCs were pre-treated with DMSO or MLN120B (10 µM) for 30 min, then stimulated with Ang II (100 nM) for the indicated times. Cell extracts were subjected to immunoblotting with the indicated antibodies. Data are representative of 3 independent experiments. (**B**,**C**) VSMCs were treated with DMSO or the indicated concentrations of MLN120B for 6 days. Cell extracts were subjected to immunoblotting with the indicated antibodies. Data are representative of at least 2 independent experiments. (**D**) qPCR analysis of *Ccn2* expression in SRAs lysates from IKKβ^+/+^ (*n* = 5) and IKKβ^−/−^ (*n* = 5) mice infused with Ang II at 1000 ng/kg/min for 1 day. Data were quantified and presented as mean ± SEM. * *p* < 0.05, 2-tailed Student’s *t* test. RQ: Relative quantification.

To further substantiate the observed fate-shaping properties of IKKβ, we next examined this question in cultured rat VSMC. In serum-starved VSMCs, we recapitulated the inhibitory effect of the selective IKKβ inhibitor MLN120B on phosphorylation of the mTORC1 substrate ribosomal protein S6 kinase (p70S6K) in Ang II-treated cells [16] (Figure 4A). However, these short-term kinetics cannot capture the transcriptionally dependent fate-shaping processes occurring in these cells. Because cultured VSMC rapidly adopt a synthetic phenotype once adhered to plastic dishes and exposed to serum, we exposed them to MLN120B for 6 days and assessed steady-state expression levels of KLF4 and β-catenin, a recently described transcriptional coactivator involved in the polarization of VSMCs toward degradative macrophage-like phenotype [37]. Treatment with MLN120B reduced the expression of both phenotypic markers in a concentration-dependent manner (Figure 4B). Furthermore, IKKβ inhibition reduced the expression of cellular communication network factor 2 (CCN2; also known as CTGF) in vitro and in vivo (Figure 4C,D), a matricellular protein linked to various fibrotic processes and recently proposed to decrease VSMCs adhesion to structural ECM, thereby promoting aortic dissections [32]. Altogether, our results identify IKKβ as a novel VSMCs fate-shaping regulator that controls early aortic remodeling events, leading to aneurysms and ruptures.

### 3.4. IKKβ Expression in Vascular Smooth Muscle Cells Is a Critical Mediator of AAA Development and Rupture

**Figure 5:**
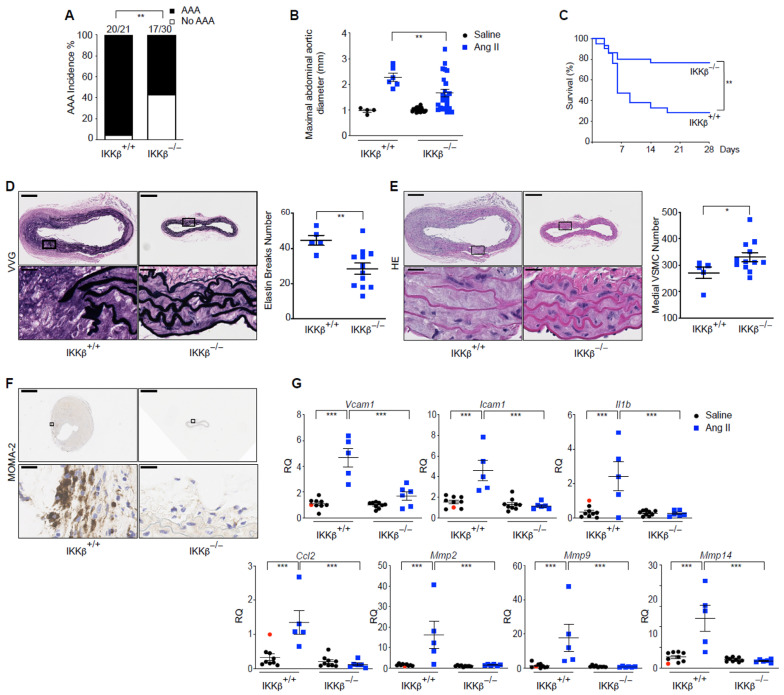
IKKβ deletion in VSMCs prevents AAA development and fatal ruptures. (**A**) AAA incidence in mice from both groups infused with Ang II at the protocol endpoint (28 days). ** *p* < 0.01, Fisher’s exact test. (**B**) Maximal abdominal aortic diameter in mice from both groups infused with saline or Ang II at the protocol endpoint (28 days). Data were quantified and presented as mean ± SEM. ** *p* < 0.01, 2-way ANOVA with Tukey’s multiple-comparison test. (**C**) Survival curve of IKKβ^+/+^ (*n* = 21) and IKKβ^−/−^ (*n* = 30) mice infused with Ang II for 28 days. ** *p* < 0.01, Log-rank (Mantel–Cox) test. (**D**) Representative Verhoeff–Van Gieson (VVG) stains of SRAs from IKKβ^+/+^ (*n* = 5) and IKKβ^−/−^ (*n* = 12) mice infused with Ang II, with elastin break counts. Scale bars: 150 µm; magnification scale bars: 37.5 µm. Data were quantified and presented as mean ± SEM. ** *p* < 0.01, 2-tailed Student’s *t* test. (**E**) Representative hematoxylin and eosin (HE) stains of SRAs from IKKβ^+/+^ (*n* = 5) and IKKβ^−/−^ (*n* = 12) mice infused with Ang II, with medial VSMC counts. Scale bars: 150 µm; magnification scale bars: 37.5 µm. Data were quantified and presented as mean ± SEM. * *p* < 0.05, 2-tailed Student’s *t* test. (**F**) Representative MOMA-2 histochemistry of SRAs from IKKβ^+/+^ (*n* = 5) and IKKβ^−/−^ (*n* = 12) mice infused with Ang II. Scale bars: 600 µm; magnification scale bars: 37.5 µm. (**G**) qPCR analysis of indicated mRNA expression in adSRAs lysates from IKKβ^+/+^ (*n* = 9) and IKKβ^−/−^ (*n* = 9) mice infused with saline, and from IKKβ^+/+^ (*n* = 5) and IKKβ^−/−^ (*n* = 6) mice infused with Ang II at the protocol endpoint. Red circles represent the reference. Data were quantified and presented as mean ± SEM. *** *p* < 0.001, 2-way ANOVA with Tukey’s multiple-comparison test. RQ: Relative quantification.

Given the crucial roles of IKKβ in orchestrating inflammatory responses, mediating VSMCs phenotypic switching, and driving early anabolic processes that contribute to aortic wall remodeling and degradation, we assessed the involvement of VSMCs IKKβ in AAA development and subsequent fatal ruptures. Systemic Ang II infusion at 1000 ng/kg/min successfully induced AAA in hypercholesterolemic mice, and deletion of IKKβ in VSMCs dramatically reduced the incidence and maximal abdominal aortic diameters of aortic SRAs while increasing animal survival. (Figure 5A–C). Significantly, fatalities due to aortic ruptures were diminished by ~48% in the IKKβ^−/−^ group (Supplementary Figure S4A,B). Next, SRAs isolated from animals that survived the 28 days were further analyzed for classical endpoints of aortic wall remodeling and inflammation. Elastin degradation and adventitial macrophage recruitment were significantly reduced in the absence of IKKβ in the media, effects that corresponded to healthier medial regions composed of densely populated, multilayered SMCs (Figure 5D–F). Furthermore, adjacent aortic tissues to the SRAs (adSRAs) were isolated from the randomized animals described above and used to extract RNA and interrogate mRNA expression associated with aortic disease. In Ang II-treated animals, the aortas showed an almost total dependence on IKKβ in medial VSMCs for the induction of classical inflammatory mRNA transcripts, including cytokine/chemokine (interleukin-1β (*Il1b*)/C-C motif chemokine ligand 2 (*Ccl2*)), adhesion molecules that regulate leukocyte recruitment (vascular cell adhesion molecule 1 (*Vcam1*)/intercellular adhesion molecule 1 (*Icam1*)), and ECM remodeling enzymes (matrix metalloproteinase 2 (*Mmp2*), 9 (*Mmp9*) and 14 (*Mmp14*)) (Figure 5G).

Although IKKβ deficiency in the vascular wall reduced AAA development and fatal ruptures over 28 days of Ang II infusion, up to 57% of animals still developed AAA lesions (Figure 5A and Figure S4A,B). Thus, we next tested whether a lower dose of Ang II in our mouse model would demonstrate the absolute requirement for IKKβ expressed in VSMCs for AAA development and its complications. Interestingly, animals lacking IKKβ expression in the vascular wall and treated with 500 ng/min/kg of Ang II for 28 days showed no signs of aneurysm, whereas the control group had 40% (6/15) of IKKβ^+/+^ mice developing AAA, with 20% Type I lesions (dilated lumen in the supra-renal region of the aorta with no thrombus), 13% Type IV lesions (a form in which there are multiple aneurysms containing thrombus, some overlapping, in the SRAs of the aorta), and 7% (1/15) mortality due to fatal abdominal rupture (Supplementary Figure S4B–E). Preservation of the elastin network and multilayered medial SMCs, reduced MMP2 protein in the media of SRAs, and a significant inhibitory effect on selected inflammatory transcripts in aorta homogenates paralleled the absence of aortopathy (Supplementary Figure S4F–I). Levels of circulating neutrophil/macrophage chemokines known to be present in human AAA and murine models were also significantly reduced in the VSMCs-specific *Ikbkb*-knockout mice (Supplementary Figure S4J). Taken together, the results suggest that the inflammatory function of IKKβ within the vascular wall is required for the recruitment of immune cells, production of MMPs by infiltrating leukocytes and resident VSMCs, and the breakdown of the elastin network, which ultimately leads to vascular wall remodeling events associated with the presence of aneurysms and ruptures in this model [32,40].

### 3.5. Immune System Cells in Human AAA Show Increased IKKβ Expression, and Inhibition of IKKβ Reduces Human VSMCs Transdifferentiation into Macrophage-like Cells

**Figure 6:**
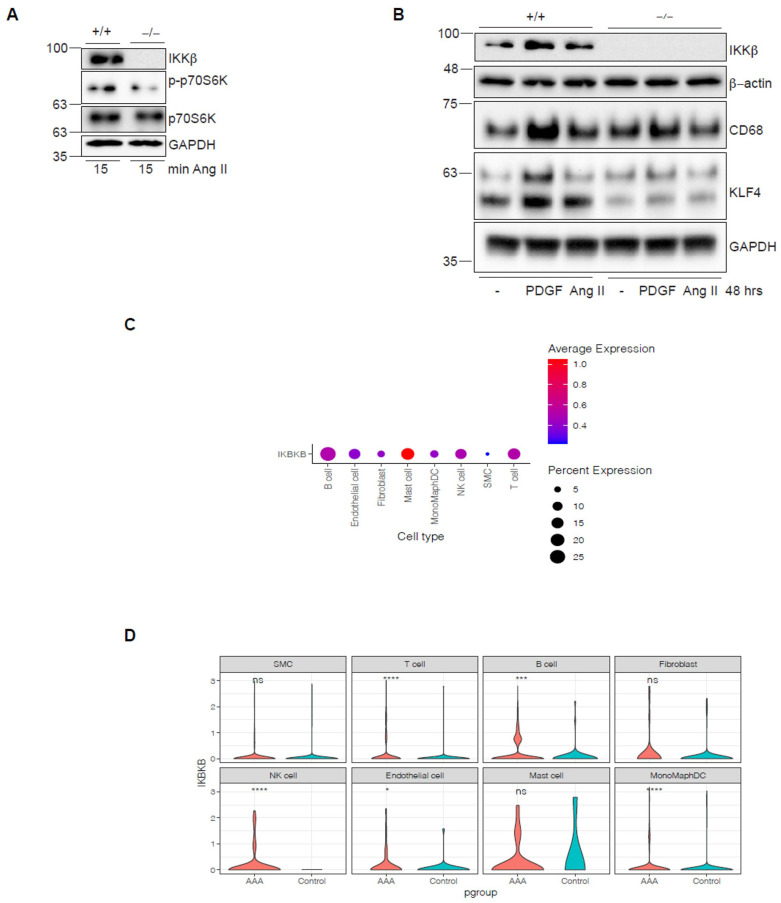
Deletion of IKKβ in HVSMCs restricts their transdifferentiation into macrophage-like cells, whereas IKKβ is expressed in immune cells from human AAA. (**A**) Indicated quiescent HVSMCs were treated with 1 μM Ang II for 15 min. Cell extracts were subjected to immunoblotting with the indicated antibodies. Data are representative of 2 independent experiments. (**B**) Indicated HVSMCs were treated with 1 μM Ang II or 10 ng/μL PDGF-BB for 48 h. Cell extracts were subjected to immunoblotting with the indicated antibodies. Data are representative of 2 independent experiments. (**C**,**D**) scRNA-seq analysis of human AAA (*n* = 4) and nonaneurysmal (*n* = 2) control samples shows relative *IKBKB* expression across all cell populations, presented as a dot plot (**C**) and a violin plot (**D**). Gene expression distributions are presented by violin plots. * *p* < 0.05, *** *p* < 0.001, **** *p* < 0.0001. 2-tailed Student’s *t*-test.

Our data showed that IKKβ removal in vivo and inhibition with MLN120B in rodent VSMCs had protective effects, reducing the inflammatory response and preserving the contractile phenotype over the degradative VSMCs phenotype, without an anabolic signature. However, it remains unclear whether these findings would translate to human VSMCs (HVSMCs). Ang II stimulation failed to induce phosphorylation of the mTORC1 substrate p70S6K in a CRISPR/Cas9-engineered population of primary HVSMCs deficient in IKKβ, as previously shown [16] (Figure 6A). Importantly, as observed in rodent VSMCs above, IKKβ inhibition in primary HVSMCs decreased expression of both KLF4 and the CD68 macrophage marker in response to Ang II or the classic phenotypic inducer PDGF-BB [41] (Figure 6B).

Lastly, we performed single-cell RNA sequencing (scRNA-seq) on human AAA samples and compared them with those from atherosclerotic controls. Analysis of human AAA samples showed reduced HVSMCs abundance relative to other immune cell populations, making a direct comparison of IKKβ expression with controls challenging and revealing no significant difference in IKKβ expression in HVSMCs (Figure 6C,D). Nevertheless, IKKβ expression is significantly upregulated in human AAA across all immune cell populations, including T cells, B cells, NK cells, and macrophage/monocyte populations. It is also considerably upregulated in endothelial cells and fibroblasts (Figure 6D). Notably, our in vivo and in vitro observations support a polarization of VSMCs towards a macrophage-like degradative phenotype in aortic diseases [37], which may contribute to the marked rise in IKKβ expression within macrophage/monocyte populations and the reduced detection of HVSMCs in scRNA-seq. It could also help explain the enhanced recruitment of other immune cells, including B, T, and NK cells.

## 4. Discussion

In the present study, we first demonstrate that transcriptomic changes associated with long-term adverse outcomes of AAA are evident as early as 24 h after Ang II infusion. Moreover, we show that the earliest events underlying AAA pathophysiology involve activation of anabolic pathways rather than inflammatory pathways. Furthermore, in addition to directly activating the mTORC1 signaling cascade [16] and modulating early anabolic events, IKKβ plays a crucial role in the phenotypic switching of VSMCs towards a degradative phenotype. Collectively, our study identifies a novel regulatory signaling axis, the IKKβ-mTORC1 axis, in the development and progression of AAA. We propose that controlling the anabolic response through IKKβ warrants consideration in the design of future studies aimed at identifying and characterizing potential therapeutic targets for early AAA lesions. From a clinical perspective, an association between anabolic events and aortic diseases has been suggested. Notably, anabolic reactions have been observed to increase with advancing age [6], a predominant clinical characteristic of AAA. Furthermore, recent case reports have established a potential correlation between the use of anabolic steroids and the occurrence of thoracoabdominal aneurysms and aortic dissection [42,43].

For more than two decades, studies have used Ang II infusion in hyperlipidemic mouse models to gain mechanistic insights into human AAA [27]. In this context, Ang II is considered the primary inflammatory trigger, initiating a complex response in which cytokines and MMPs produced by resident fibroblasts, VSMCs, endothelial cells, and innate/adaptive immune cells drive the deleterious remodeling of the vascular wall [44]. In addition to this inflammatory component, accumulating evidence indicates that VSMCs within remodeled vascular regions exhibit notable plasticity and can adopt various phenotypes, with potential clinical implications that may be beneficial or detrimental [45]. Understanding the distinct stages and mechanisms of VSMCs activation, including transitions from quiescence to invasion of remodeled vascular areas and fate diversification, is crucial for devising therapeutic interventions aimed at mitigating pathological VSMCs behaviors while preserving or enhancing their stabilizing functions. In this study, using a highly characterized Ang II-infused *ApoE*-deficient mouse model, we identified an early molecular anabolic signature associated with mTORC1 pathway activation (Figure 1).

Dysregulation of mTORC1 activation was recently shown to promote aortopathy by inducing phenotypic switching of VSMCs to a degradative phenotype, thereby driving proteolysis, extracellular matrix degeneration, and inflammation in the aortic wall [37]. While blocking mTOR signaling is a promising therapeutic target for limiting both thoracic aortic aneurysm and AAA development [46,47,48], a recent study indicated that targeting mTORC1 with rapamycin promotes aortic dissection [32]. Interestingly, this was associated with continuous, mTORC1-independent induction of CCN2 by Ang II, which decreased VSMCs adhesion to structural ECM and allowed extensive dissection [32]. Rapamycin-induced aortic dissection could also be related to the action of the inhibitor in other cell types. In the current study, we show that targeting upstream of mTORC1 (i.e., conditional deletion of IKKβ in VSMCs) reduces aortic dilatation and fatal ruptures. Based on our detailed analysis of the SRAs in early (one to four days) or late (28 days) infusion protocols, we propose that IKKβ plays pivotal roles in initiation and disease progression through at least three distinct mechanisms: (1) by controlling mTORC1 in VSMCs exposed to Ang II, IKKβ rapidly engages anabolic events leading to (A) the generation of macrophage-like VSMC responsible for early vascular wall remodeling events, as well as (B) rapid metabolomic reprogramming towards OXPHOS; (2) in combination with increased ribosome synthesis and the UPR, these events create conditions favoring ROS production [49,50] and an exacerbated inflammatory response in the vascular wall, leading to (3) leukocyte infiltration and sustained NF-κB-dependent induction of *Ccn2* [51], culminating in aortic dissections and fatal ruptures. Rapid monocyte recruitment [25] and/or the presence of sparse tissue-resident macrophages could contribute to rapid vascular wall remodeling. However, the early appearance of the mTORC1 signature (24 h) alongside the VSMCs switch toward a degradative phenotype suggests a direct role of IKKβ in these effector cells. Importantly, IKKβ might also have other roles in VSMCs and macrophages, as recently demonstrated [5,52].

Phenotypic modulation of VSMCs occurs early in aneurysm development in both animal models and human AAA samples, and this switch is crucial to AAA development. In healthy aortic tunica media, VSMCs exhibit a contractile phenotype, and during aortic aneurysm formation, they switch to a synthetic/proliferative phenotype. Recently, single-cell RNA sequencing of human AAA samples showed that the VSMCs phenotypic switch is not a single transition but involves multiple VSMCs phenotypes, including T-cell-like, macrophage-like, and mesenchymal-like, and that these VSMCs play pivotal roles in disease development [53]. It remains unclear whether these phenotypically altered VSMCs can also mimic immune cell phenotypes recruited to the AAA site. Here, we show that targeting IKKβ specifically in VSMCs resulted in a decreased number of macrophages and leucocytes and preserved the VSMCs contractile phenotype. These findings provide valuable mechanistic insight into the role of IKKβ in rapid vascular wall remodeling. However, detailed studies are required to decipher the roles of IKKβ in VSMCs differentiation into specific subtypes and to determine whether the decreased number of immune cells observed in AAA in IKKβ^−/−^ animals is due to reduced recruitment from the blood circulation or to VSMCs that have switched to macrophage-like VSMCs.

Through its substrates and interactors, including SREBP, eIF4E-binding protein 1/2 (4E-BP1/2), ribosomal protein S6 kinases 1/2 (p70S6K1/2), the Ser/Thr kinase ULK1, the transcriptional co-activator PGC-1α, and the transcription factor YY-1, the effector anabolic kinase complex mTORC1 controls the synthesis of several essential macromolecules (lipids, proteins, ribosomal RNAs) and directly influences cellular energy metabolism by inhibiting autophagy and/or increasing mitochondrial function/biogenesis [6]. Although essential for cell growth, mTORC1 activation is also linked to aging (decreased longevity) [6] and fibrosis [31], processes that play major roles in AAA development. We propose that the dramatic reduction in aortic dilatation and fatal ruptures observed in IKKβ^−/−^ animals is explained by IKKβ acting as a molecular hub in the vascular wall, allowing vasoactive molecules such as Ang II to control both the classical inflammatory effectors, such as NF-κB and mTORC1. In this context, it would be interesting to determine whether the failure of the vasoactive agent norepinephrine to promote AAA is related to its inability to stimulate the IKKβ-mTORC1-dependent anabolic/inflammatory responses [54].

## 5. Limitations

Although RNA-Seq data were analyzed, we acknowledge that our study does not show IKKβ protein expression or enzymatic activation in the context of human AAA, and that future studies aimed at precisely addressing this in medial VSMCs are essential. It is, however, interesting to note that increased IKKβ protein expression has previously been observed in human aortas derived from advanced atherosclerotic lesions [55]. This might partly explain why we observed no difference in *IKBKB* mRNA abundance in VSMCs derived from AAA compared to non-aneurysmal (atherosclerotic) patients (Figure 6C,D). In addition, our survival studies show that mortality exceeds 50% 7 days after Ang II infusion and increases to 70% between 14 and 21 days following Ang II infusion (Figure 5C), which is markedly different from what is typically reported for this widely used *ApoE^−/−^*/Ang II model [56]. However, these findings are in line with other VSMCs-CreER-expressing studies [11,57]. CreER exhibits higher toxicity than Cre [58]. Moreover, Cre toxicity has been reported in the cardiovascular system [59,60] and could therefore explain the increased mortality rate observed in these CreER^T2^ models. Importantly, because we used SMA-CreER^T2^- *ApoE^−/−^* as controls in our study, our results are conclusive that the absence of IKKβ in VSMCs prevents aortic dilatation and fatal rupture.

## 6. Conclusions

Our study identified a previously unrecognized role for IKKβ in AAA development and progression, highlighting a novel therapeutic target. However, prolonged systemic IKKβ inhibition has context-dependent effects on the immune system and inflammatory processes [61]. Therefore, further investigation is needed to determine the effects of local or systemic IKKβ inhibition in humans with AAA.

## Figures and Tables

**Table 1:** AAA classification in both groups infused with saline (Day 0) and Ang II 1000 ng/kg/min for 2 to 8 days (6 animals/group/day).

0	Type	Day 0	Day 2	Day 3	Day 4	Day 8
IKKβ^+/+^		100%	50%	33%	0%	17%
					
IKKβ^−/−^		100%	100%	83%	83%	50%
IKKβ^+/+^	I	0%	0%	0%	0%	0%
					
IKKβ^−/−^		0%	0%	0%	0%	0%
IKKβ^+/+^	II	0%	17%	33%	50%	50%
					
IKKβ^−/−^		0%	0%	17%	17%	33%
IKKβ^+/+^	III	0%	0%	0%	0%	0%
					
IKKβ^−/−^		0%	0%	0%	0%	0%
IKKβ^+/+^	IV	0%	33%	0%	33%	17%
					
IKKβ^−/−^		0%	0%	0%	0%	0%
IKKβ^+/+^	Rupture	0%	0%	33%	17%	17%
					
IKKβ^−/−^		0%	0%	0%	0%	17%

## Data Availability

The data supporting the findings of this study are available from the corresponding author upon reasonable request. The RNA sequencing (RNA-Seq) data generated in this study have been deposited in NCBI GEO (accession number: GSE265897; key: ijkdsqasrdkdnyj).

## Supplementary Materials

The following supporting information can be downloaded at: https://www.mdpi.com/article/10.3390/cells15030218/s1, Table S1: List of antibodies used in the IHC and IF staining. Table S2: Sequences of the primers and UPL probes. Figure S1: Biochemical and pathophysiological characteristics of the IKKβ^+/+^ and IKKβ^−/−^ mice and ROSA26/lacZ reporter mouse line. (A) Schematic of the enzymatic steps in cholesterol biosynthesis and the induction of associated transcripts by Ang II in wild-type mice after 24 h of treatment. Bubble size is proportional to fold change (Log2), and colour indicates significance (−log10 *p*-value). For clarity, only selected metabolic intermediates are shown. (B) Activation of SMA-CreER^T2^ upon tamoxifen injection is restricted to VSMC. Eight-week-old heterozygous SMA-CreER^T2^ROSA26/lacZ^+/−^ male mice were treated with either 1 mg tamoxifen (left panel) or vehicle (peanut oil; right panel) for 5 days. Isolated SRAs were collected and subjected to X-Gal staining of 10 µm cryosections. Arrows: elastic fibres; arrowheads: endothelial cells; al: aortic lumen; m: media. Scale bars: 40 µm. (C) Representative haematoxylin and eosin (HE) stains of SRAs from IKKβ^+/+^ (*n* = 2) and IKKβ^−/−^ (*n* = 2) mice infused with saline for 28 days. Scale bars: 150 µm. (D) Endpoint systolic blood pressure was measured during the last week of Ang II infusion at 1000 ng/kg/min for 28 days. Body weights were measured at the protocol endpoint. Data were quantified and presented as mean ± SEM. (E) Total plasma cholesterol, very-low-density lipoprotein (VLDL) (FPLC fractions 7–16), low-density lipoprotein (LDL) (fractions 17–32), and high-density lipoprotein (HDL) (fractions 33–50) cholesterol were measured at the endpoint of Ang II infusion at 1000 ng/kg/min for 28 days. Data represent pooled plasma from 4 mice per group. Plasma triglyceride concentration was quantified in individual mice (*n* = 4/group) and presented as mean ± standard deviation. Figure S2: VSMC-specific IKKβ deletion in mice preserves their contractile phenotype. (A) SRAs from IKKβ ^+/+^ (*n* = 5) and IKKβ ^−/−^ (*n* = 5) mice were pooled, and proteins were extracted to validate IKKβ deletion. (B) Proteins were extracted from SRAs of IKKβ ^+/+^ (*n* = 11) and IKKβ ^−/−^ (*n* = 11) mice treated with Ang II for 28 days or that died suddenly from aortic rupture. Tissue extracts were subjected to immunoblotting with the indicated antibodies. For markers run on gels in which α-SMA was detected, the β-actin loading controls shown in the figure were obtained from separate membranes with identical samples, processed in parallel. This approach was necessary because the use of the α-SMA antibody altered the membrane, which could not be stripped properly. C) Data are presented as mean ± SEM. * *p* < 0.05, ** *p* < 0.01, **** *p* < 0.0001, 2-tailed Student’s *t* test. Figure S3: IKKβ^−/−^ mice still respond to Ang II. (A) Volcano plot of differentially expressed transcripts (DET) in the SRAs of IKKβ^−/−^ mice after 24 h of Ang II treatment. Each point shows the mean fold change and the associated *p*-adjusted value for a single transcript, comparing Ang II (*n* = 5 mice) with saline (*n* = 4 mice). Coloured numbers indicate the number of significantly induced (red) and reduced (blue) transcripts at the given threshold (dotted lines), as described in Materials and Methods. (B) Heatmap of significant DET. Expression fold changes have been transformed to z-scores, and clustering has been performed on rows (mice) and columns (transcripts). Figure S4: IKKβ expression in vascular SMC is necessary for AAA development and fatal rupture. (A) Cause of death in both groups infused with Ang II at 1000 ng/kg/min for 28 days. TAA: thoracic aortic aneurysm. (B) AAA classification in both groups infused with Ang II at 500 ng/kg/min or 1000 ng/kg/min for 28 days. (C) Representative histochemistry of conditional IKKβ deletion in VSMC of SRAs from IKKβ^+/+^ (*n* = 2) and IKKβ^−/−^ (*n* = 2) mice infused with 500 ng/kg/min of Ang II for 28 days. Black arrow and red arrow indicate intimal and adventitial staining, respectively, demonstrating specific IKKβ knockout in the media. Scale bars: 600 µm; magnification scale bars: 37.5 µm. (D) AAA incidence in mice from both groups infused with Ang II at the protocol endpoint. * *p* < 0.05, Fisher’s exact test. (E) Maximal abdominal aortic diameter in mice from both groups infused with saline or Ang II at the protocol endpoint. Data were quantified and presented as mean ± SEM. ** *p* < 0.01, 2-way ANOVA with Tukey’s multiple-comparison test. (F) Representative Verhoeff–Van Gieson (VVG) stains of SRAs from IKKβ^+/+^ (*n* = 8) and IKKβ^−/−^ (*n* = 7) mice infused with Ang II, with elastin breaks counted. Scale bars: 150 µm; magnification scale bars: 37.5 µm. Data were quantified and presented as mean ± SEM. ** *p* < 0.01, 2-tailed Student’s *t* test. (G) Representative haematoxylin and eosin (HE) stains of SRAs from both IKKβ^+/+^ (*n* = 8) and IKKβ^−/−^ (*n* = 7) mice infused with Ang II, with medial VSMC count. Scale bars: 150 µm; magnification scale bars: 37.5 µm. Data were quantified and presented as mean ± SEM. ** *p* < 0.01, 2-tailed Student’s *t* test. (H) Representative MMP2 immunohistochemistry of SRAs from IKKβ^+/+^ (*n* = 8) and IKKβ^−/−^ (*n* = 7) mice infused with Ang II, with scoring. Scale bars: 150 µm; magnification scale bars: 37.5 µm. Data were quantified and presented as mean ± SEM. ** *p* < 0.01, 2-tailed Student’s *t* test. (I) qPCR analysis of *Vcam1* and *Mmp2* mRNA expression in adSRAs lysates from IKKβ^+/+^ (*n* = 9) and IKKβ^−/−^ (*n* = 9) mice infused with saline, and from IKKβ^+/+^ (*n* = 11) and IKKβ^−/−^ (*n* = 13) mice infused with Ang II at the protocol endpoint. Red circles represent the reference. Data were quantified and presented as mean ± SEM. * *p* < 0.05, *** *p* < 0.001, 2-way ANOVA with Tukey’s multiple-comparison test. RQ: relative quantification. (J) Levels of circulating neutrophil/macrophage chemokines from both groups infused with Ang II (*n* = 5). Data were quantified and presented as mean ± SEM. * *p* < 0.05, ** *p* < 0.01, 2-tailed Student’s *t* test. References [62,63,64,65,66,67,68,69,70,71,72,73,74,75,76,77] are cited in Supplementary Materials.

## Abbreviations

The following abbreviations are used in this manuscript:

AAA	abdominal aortic aneurysm
ECM	extracellular matrix
VSMCs	vascular smooth muscle cells
IKKβ	inhibitor of nuclear factor kappa B kinase subunit beta
NF-kB	nuclear factor kappa B
Ang II	angiotensin II
ApoE	apolipoprotein E
SRAs	suprarenal abdominal aortas
RNA-seq	RNA sequencing
DET	differentially expressed transcripts
GSEAs	Gene Set Enrichment Analyses
mTORC1	mammalian target of rapamycin complex 1
UPR	unfolded protein response
TFBS	transcription factor binding sites
KLF	Krüppel-like factors
MMP	matrix metalloproteinase
CCN2	cellular communication network factor 2
TET2	ten-eleven translocation-2
GAL3	galectin-3
TNF-α	tumor necrosis factor-alpha
HVSMCs	human VSMCs
