# Supplementary material for: Early IKKβ-Dependent Anabolic Signature Governs Vascular Smooth Muscle Cells Fate and Abdominal Aortic Aneurysm Development
Source: Cells. 2026 Jan 23;15(3):218. doi: 10.3390/cells15030218 (PMC12896606; doi:10.3390/cells15030218)
Supplement: Supplementary file 1 [file cells-15-00218-s001.zip › cells-4085889 supplementary.pdf]

SUPPLEMENTAL MATERIAL

Early IKKβ-dependent anabolic signature governs vascular smooth muscle cells fate and abdominal aortic aneurysm development

Priscilla Doyon[1*], Ozge Kizilay Mancini[2*], Florence Dô[1,3], David Huynh[3], Gaétan Mayer[1,4], Stéphanie Lehoux[5], Huy Ong[1], Maelle Batardière[3], Vincent Quoc-Huy Trinh[3], Ying Wen[6], Wai Ho Tang[6], Sylvie Marleau[1], Simon-Pierre Gravel[1], and Marc J Servant[1]**

[1] Faculty of Pharmacy, Université de Montréal, Montréal, Canada

[2] Diagnostic and Molecular Pathology, McGill University, Montreal, Quebec, Canada.

[3] Institute for Research in Immunology and Cancer, Université de Montréal, Montréal, Canada

[4] The Montreal Heart Institute, Université de Montréal, Montréal, Canada

[5] Lady Davis Institute for Medical Research, McGill University, Montréal, Canada

[6] Institute of Pediatrics, Guangzhou Women and Children's Medical Centre, Guangzhou Medical University, China.

* Contributed equally

** Corresponding author. e-mail: marc.servant@umontreal.ca

SUPPLEMENTARY METHOD

Animal Experiments:

All animal experiments were conducted in a specific pathogen-free barrier facility in accordance with institutional guidelines and local protocols (18-097 and 18-098), approved by the Ethics Committee of the Université de Montréal, and in accordance with the European Communities Council Directive 2010/63/EU on the protection of animals used for experimental purposes.

Mouse models

For these investigations, we used the Angiotensin II (Ang II) infusion technique, blood pressure monitoring, and aneurysm characterization. We employed the Ang II-infused apolipoprotein $E^{-/-}$ *(ApoE*$^{-/-}$) C57BL/6J mouse model, initially developed by Daugherty et al. [62]. This model typically develops suprarenal (SRA) abdominal aortic aneurysm (AAA) in approximately 80–85% of cases. However, the incidence of AAAs in female *ApoE*$^{-/-}$ mice infused with Ang II is relatively low (around 20%). Therefore, our focus was on male *ApoE*$^{-/-}$ mice, which exhibit a significantly higher AAA incidence, ranging from 80 to 100% [63], and on the known crosstalk between nuclear factor kappa B (NF-κB) and estrogen signaling. Nevertheless, the authors acknowledge that focusing solely on one sex presents limitations, as sex differences have been extensively documented in mouse studies. Mice (n=231) were fed a regular chow diet and used at 8 weeks of age. For this study, transgenic mice were crossed onto a C57BL/6J background >10 times. The *Ikbkb* conditional knockout mouse model in a hyperlipidemic genetic background was generated by crossing mice with a tamoxifen-inducible Cre-loxP system restricted to vascular smooth muscle cells (VSMC) (Cre driven by the smooth muscle α-actin (SMA) promoter *Acta2*; graciously obtained by Drs. Pierre Chambon and Daniel Metzger (IGBMC, France) as previously shown [64]) with mice homozygous for the floxed *Ikbkβ* allele (mice received from Dr. Michael Karin, UCSD, USA) into the *ApoE*$^{-/-}$ hyperlipidemic background (Jackson Lab; stock #: 002052). This produced the transgenic homozygous mouse model referred to as SMA-

CreER^{T2}Ikkβ$^{Flox/Flox}$*ApoE*$^{-/-,}$ in which the inhibitor of nuclear factor kappa B kinase subunit beta (IKKβ) expression can be temporally and selectively prevented in *ApoE*$^{-/-}$ VSMC on a C57BL6 background. A heterozygous SMA-CreER^{T2}ROSA26/lacZ$^{+/-}$ mouse line was also generated by mating SMA-CreER^{T2} animals with the ROSA26/lacZ *reporter mouse line* (Jackson Lab; stock #003474), which contains a *loxP*-flanked DNA STOP sequence preventing expression of the downstream *lacZ* gene, which encodes β-galactosidase. Male SMA-CreER^{T2}Ikkβ$^{Flox/Flox}$ApoE$^{-/-}$ are referred to as IKKβ$^{-/-}$, and their control littermates SMA-CreERT2ApoE$^{-/-}$ are referred to as IKKβ$^{+/+}$. When indicated, animals were injected intraperitoneally (i.p.) with tamoxifen (1 mg/day) for 5 days. After 7 days of rest, mice were subjected to saline or Ang II infusion.

Tamoxifen treatment and osmotic pump implantation

Eight-week-old male mice received a daily intraperitoneal injection of 1 mg (100 μL) tamoxifen for five consecutive days. Injection sites alternated between the left and right sides. Tamoxifen was prepared as a 10 mg/mL stock solution (100 mg tamoxifen-free base (Sigma) in 0.5 mL ethanol, diluted with 9.5 mL peanut oil). No injection-site inflammation was observed in 231 male mice. A seven- to nine-day recovery period was allowed for genetic recombination. Twelve to fourteen days after tamoxifen injection, osmotic minipumps (Alzet pump, 100 μL, #1004) delivering Angiotensin II (Ang II, Sigma-Aldrich, A9525) over 1, 2, 4, 8, or 28 days were implanted. Mice were anesthetized with 3% isoflurane/oxygen (2 L/min, 100% O_2). One hour prior, mice received a subcutaneous injection of 10 mg/kg carprofen (50 mg/ml), diluted 1:100 with saline, then further diluted with 37°C sterile saline to a total volume of 1 mL to maintain hydration and circulating volume. A lubricant gel was applied to the corneas. Mice were placed under the anesthesia mask on a pre-heated platform, with isoflurane reduced to 2% and oxygen to 1 L/min to avoid deep anesthesia. The surgical site was shaved and disinfected (three alternating alcohol 70%/iodine passes). The absence of the palpebral reflex or withdrawal response

confirmed the depth of anesthesia. Small (maximum 1 cm) incisions were made between the scapulae for minipump implantation (1.5 cm length, 0.6 cm diameter). The incisions were closed with up to two Michel clips (sutures were used in case of complications). A thin layer of lidocaine/flamazine mixture was applied to the wound before awakening. Post-surgery, cages were placed on heating pads until the mice recovered. Animals were returned to their cages (with pre-fabricated nests) and monitored for pain or discomfort for the first four hours. For the following two days, mice received postoperative analgesia with carprofen (10 mg/kg) once daily (SID). Clips were removed after 10 days. Animals exhibiting pain or discomfort beyond 5 days received additional analgesia or were euthanized with CO_2 followed by cervical dislocation.

In situ examination and AAA scoring

Animals were sacrificed 1, 2, 4, 8, or 28 days after minipump implantation for organ harvesting. Mice were anesthetized with 2–3% isoflurane (pedal reflex absence confirmed) and euthanized by exsanguination. Cardiac puncture with a 25G 5/8 needle was performed to collect approximately 1.5 mL of blood for circulating pro-inflammatory cytokine analysis. Organ harvest followed cardiac puncture and pneumothorax. A midline incision was made from the abdomen to the chest. Saline was used to flush the circulation, after which the internal organs were removed and access to the retroperitoneum was gained. Using a Zeiss Discovery.V8 microscope and a Canon camera, the SRA abdominal aorta was examined for haematomas. The fresh aorta was dissected and cleared of adventitial fat following gentle perfusion. Using dissection needles, the aorta was fixed to a black wax plate in PBS. After imaging each aorta with a Zeiss microscope, the maximum abdominal aortic diameter was measured using Image-Pro Plus software (Media Cybernetics, Silver Springs, MD, USA). AAA was defined as abdominal aortic dilatation greater than 1.5 times. The severity of AAA was classified according to Daugherty [65], i.e., suprarenal dilation without thrombosis (type I), suprarenal dilation with thrombosis (type II), a pronounced bulbous form of type II (type III), and multiple aneurysms containing thrombus (type IV).

Blood pressure measurement

Blood pressure was monitored using non-invasive tail-cuff plethysmography (Kent Scientific Corporation, Torrington, USA). Blood pressure assessment began 3 days before minipump implantation to habituate the mice to the procedure and continued at three intervals (days 0, 4, and 7) over 1 week. Mice were placed on a heating platform for 10 min before blood pressure assessment. A minimum of five measurements was taken until blood pressure stabilized, and, following stabilization, a minimum of eight measurements was taken per mouse and averaged for analysis. Blood pressure was monitored by the same person at the same time of day.

Plasma cholesterol, lipoproteins and triglycerides measurements

Mouse plasma lipoprotein cholesterol profiles were obtained from 100 µL of pooled plasma from IKKβ$^{+/+}$ and IKKβ$^{-/-}$ mice (n=4) infused with Ang II at 1000 ng/kg/min for 28 days. Pooled plasma samples were injected onto a Superose 6 10/300 GL column (GE Life Sciences; Cat. #17-5172-01) and eluted with PBS (Wisent, Cat. #311-010-CL) at a flow rate of 0.1 mL/min at 4°C, mounted on an ÄKTA explorer system (GE Healthcare). Cholesterol was quantified in each collected fraction using the cholesterol E enzymatic assay from Wako (Cat. #439-17501). Plasma triglyceride (TG) concentrations were measured using an enzymatic colourimetric assay from Wako (632-50991).

Immunohistochemistry and immunofluorescence staining

SRA regions were fixed in formalin and paraffin-embedded at the Institute for Research in Immunology and Cancer (IRIC) Histology core facility (Université de Montréal) for 24 hours. Paraffin blocks were cut into 4 µm sections. Hematoxylin and eosin (HE) staining was performed to assess general morphology and VSMC count. The medial VSMC count was determined by counting the number of nuclei surrounded by the internal and external elastic lamina in duplicate. The Verhoeff–Van Gieson (VVG) stain was performed to visualize elastin fibres. Elastin degradation was quantified by

counting breaks per vessel in duplicate. Immunohistochemistry (IHC) and immunofluorescence (IF) reactions were carried out on the Bond RX Stainer (Leica Biosystems, Buffalo Grove, IL, USA). Sections were deparaffinized inside the immunostainer. Antigen retrieval was conducted using Heat-Induced Epitope Retrieval either with a citrate-based (pH 6.0) epitope retrieval solution (H1) or an EDTA-based (pH 9.0) epitope retrieval solution (H2) or using Proteolytic-Induced Epitope Retrieval with Enzyme 1 (E1) (Leica Biosystems proprietary reagent). Sections were next incubated with 150 μL of the first antibody diluted with BOND primary antibody diluent (AR9352). IHC and IF signals were acquired with specific secondary antibodies by using Bond Polymer DAB Refine kit (No. DS9800, Leica Biosystems) and Bond Research Detection System 2 (No. DS9777, Leica Biosystems), respectively, according to the manufacturer's instructions. Stained slides were coverslipped and scanned using the Hamamatsu NanoZoomer® Digital Pathology system 2HT, and the images were visualized with NDP.view2 software. Matrix metalloproteinase 2 (MMP2) staining was scored using Visio-morph, Tissuemorph Digital Pathology software. Galectin 3 (Gal3) (Alexa Fluor 488) and α-SMA (Alexa Fluor 555) co-immunofluorescence staining was analyzed with QuPath 0.5.0 software. The minimum Gal3 threshold was set to the upper limit of the elastin autofluorescence. The antibodies, dilutions, pretreatment, and treatment conditions, as well as the suppliers, are listed in **Supplemental Table S1**.

Cell culture, treatment, western blot analysis and antibodies

Primary rat aortic VSMC were isolated from Wistar rats by explant and maintained in high-glucose DMEM supplemented with 10% fetal bovine serum (FBS). Cells were identified as VSMC by morphology and immunofluorescence. All experiments were conducted on cells at passage levels 9-16. Primary human aortic smooth muscle cells (HVSMC) from ScienCell (#6110) were grown in Smooth Muscle Cells Medium (ScienCell #1101) supplemented with FBS (ScienCell #0010), smooth muscle cell growth supplement (ScienCell #1152), and penicillin/streptomycin solution (ScienCell #0503). All experiments were conducted on cells at passage levels 2-6. Quiescent VSMC and HVSMC were obtained

by incubating 95% confluent cultures in serum-free high-glucose DMEM, Ham's F-12 (1:1) supplemented with 15 mM Hepes (pH 7.4), 0.1% low-endotoxin bovine serum albumin, and 5 µg/mL transferrin for 48 hours. Cultures were maintained at 37°C in an atmosphere of 95% air and 5% CO2. For VSMC experiments using Ang II (Sigma-Aldrich; A9525) and the selective IKKβ inhibitor MLN120B (Selleckchem, #S7736), cells were treated with vehicle alone or with 10 µM inhibitor for 30 min before adding 100 nM Ang II for the indicated times. For VSMC experiments using MLN120B alone, cells were treated with vehicle or the indicated concentrations of the inhibitor for 6 days. For HVSMC experiments, cells in the first set were treated with 1 µM Ang II for 15 min. In the second set, they were treated with 1 µM Ang II or 10 ng/µl PDGF-BB (ThermoFisher, #100-14B) for 48 hours. Following the treatments, cells were rinsed twice with ice-cold PBS, and whole-cell extracts were prepared using Triton X-100 lysis buffer (50 mM Tris-HCl, pH 7.4, 150 mM NaCl, 50 mM sodium fluoride, 5 mM EDTA, 40 mM β-glycerophosphate, 1 mM sodium orthovanadate, 0.1 mM phenylmethylsulfonyl fluoride, 1 µM leupeptin, 1 µM pepstatin A, 1% Triton X-100, and 10% glycerol) for 30 min at 4°C. Lysates were clarified by centrifugation at 13,000 × g for 10 min, and equal amounts of protein (60 µg) were subjected to electrophoresis on 10% acrylamide gels. Proteins were then transferred electrophoretically to Hybond-C nitrocellulose membranes (Amersham Biosciences) in a buffer containing 25 mM Tris, 192 mM glycine, and 20% methanol. Immunoblot analysis for each antibody was performed following the manufacturer's instructions. Commercial antibodies were purchased from the following suppliers: phospho-p70 S6 Kinase (Thr389, #9205), anti-p70 S6 Kinase (#2708), anti-IKKβ (D30C6, #8943), phospho-NF-kappaB p65 (Ser536, #3031), anti-β-Catenin (#8480), anti-GAPDH (14C10, #2118), anti-CD68 (#86985) and anti-KLF4 (#4038) from Cell Signaling Technologies Inc.; anti-CCN2 (PA1-22376); anti-β-actin clone AC-74 (A5316) from Millipore-Sigma; anti-MMP9 (#ab38898) from Abcam; anti-TET2 (#21207-1-AP) from Proteintech; anti-alpha-SMA (#MA5-11547) from Invitrogen.

RNA isolation and quantitative RT-PCR analysis

Total RNA from tissues composed of the end of the thoracic aorta and the beginning of the SRA segments was extracted as follows. Briefly, tissues were cut into small pieces in Trizol and homogenized in a Dounce glass homogenizer with a glass pestle using an overhead stirrer. Chloroform (0.2 vol) was added to the mixture to isolate the aqueous layer, and an equal volume of 70% ethanol was then added. The RNA was then isolated using the PicoPure RNA isolation Kit (No. KIT0204, Thermo Fisher Scientific) according to the manufacturer's instructions. Total RNA from SRA region was extracted using the RNeasy Mini Kit (No. 74106, Qiagen). Briefly, tissues were cut into small pieces in RLT buffer and homogenized in a 1.5 mL Eppendorf tube with a PTFE tip/stainless steel shaft pestle using an overhead stirrer. The RNA was then isolated according to the manufacturer's instructions. RNA was quantified using a NanoPhotometer (Implen GmbH, Munich, Germany), and samples were evaluated for integrity using a 2100 Bioanalyzer (Agilent Technologies, Palo Alto, CA). RNA was reverse transcribed into cDNA with the Maxima First Strand cDNA synthesis kit with dsDNase (Thermo Fisher Scientific). Gene expression was determined using assays designed with the Universal Probe Library from Roche (www.universalprobelibrary.com). For each qPCR assay, a standard curve was generated to ensure the assay efficiency was between 90% and 110%. The QuantStudio7 qPCR instrument (Thermo Fisher Scientific) was used to detect the amplification level. All reactions were run in triplicate, and Relative mRNA expression was calculated according to the comparative threshold (C_T) formula $2^{-\Delta\Delta CT}$, where $\Delta\Delta C_T = \Delta C_T$ test sample–ΔC_T calibrator sample and $\Delta C_T = C_T$(target)–C_T(endogenous control). *Hprt* and *Actb* were used as endogenous controls. The sequences of the primers and Universal Probe Library (UPL) probes used are listed in **Supplemental Table S2**.

Protein isolation from SRA

Protein extraction from SRA tissues was performed using Thermo Scientific™ T-PER™ Tissue Protein Extraction Reagent (catalogue number 78510) with freshly added protease and phosphatase inhibitors.

Tissues were cut directly in a glass homogenizer tube with scissors, homogenized with a glass tip at a speed of ≤ 2, and the tip was rinsed with an additional 50 μL of T-PER buffer. The homogenate was sonicated for 5 s three times at 18% amplitude, centrifuged at 10,000 × g for 5 min at 4 °C, and the supernatant was collected for immunoblot analyses.

IKKβ deletion in human aortic smooth muscle cells

Stable populations of IKKβ-deficient HVSMC were generated as previously described [66].

Analysis of scRNA-Ssq data

Single-cell RNA sequencing data from human AAA (n = 4) and nonaneurysmal (n = 2) samples, available in the public Gene Ontology Database (GSE166676), were analyzed as previously described [67].

Cytokine profiling

Plasma samples from mice infused with 500 ng/kg/min of Ang II for 28 days were collected at necropsy, stored at −80°C, and shipped on dry ice to Eve Technologies (Calgary, AB, Canada). Samples were run undiluted in the mouse cytokine/chemokine array 44-Plex (MD44) discovery assay.

RNA-Sequencing

Total RNA from the SRA region of IKKβ$^{+/+}$ and IKKβ$^{-/-}$ mice infused with saline or Ang II (1000 ng/min/kg) for 24 hours was subjected to RNA-Sequencing. Transcriptome libraries were generated using the KAPA RNA HyperPrep (Roche) with poly (A) selection (Thermo Scientific). Sequencing was performed on the Illumina NextSeq500, yielding approximately 20M single-end 84 bp reads per sample. Sequences were trimmed for sequencing adapters and low-quality 3' bases using Trimmomatic version 0.35 [68] and aligned to the reference mouse genome version GRCm38 (gene annotation from

Gencode version M25, based on Ensembl 100) using STAR version 2.7.1a [69]. Gene expressions were obtained from STAR both as readcounts and TPM values as well as computed using RSEM [70] to obtain normalized gene and transcript level expression, in TPM values, for these stranded RNA libraries. DESeq2 version 1.30.1 [71] was then used to normalize gene read counts. Principal component analysis (PCA) showed good separation of the 4 groups (IKKβ$^{+/+}$-saline; IKKβ$^{+/+}$-Ang II; IKKβ$^{-/-}$-saline; IKKβ$^{-/-}$-Ang II), with PC1 at 18% variance and PC2 at 17% variance.

Functional classification and gene set enrichment analyses

For the volcano-plot representation of differentially expressed transcripts (DET) between groups of mice treated with Ang II (1000 ng/min/kg, 24 hours) versus controls (saline, 24 hours), threshold values for fold change > 1.2 fold (Log2 > 0.26303441) and false-discovery rate (FDR)-adjusted p-values < 0.1 (-Log10 > 1.0) were used. For the heatmap representation of DET and functional analyses, the fold change threshold was further increased at > 1.5 fold (Log2 > 0.5849265). Functional classification of DET was done with Metascape [72] using the GO Biological Processes (GOBP) and KEGG Pathway ontology sources. All genes in the genome have been used as the enrichment background. Terms with a p-value < 0.01, a minimum count of 3, and an enrichment factor > 1.5 were collected and grouped into clusters based on their membership similarities. More specifically, p-values are calculated based on the cumulative hypergeometric distribution. Enrichment and bubble graphs were generated from MetaScape data with the R software using ggplot2. Gene set enrichment analyses (GSEA) were done using GSEA software (v. 4.3.0) [29, 73] using normalized read counts, excluding undetected transcripts (absence in all samples). MSigDB gene set collections used were Hallmarks, GOBP, and Wikipathways. The Cytoscape software (v. 3.10.1) [74] with the EnrichmentMap Cytoscape App [75] was used for GSEA (GOBP) network representation. Cutoff parameters were: p-value of 0.05 and FDR q-value of 0.1. Small groups were excluded (e.g. 4 nodes or less). For leading edge analyses, heatmaps representing the fold change between Ang II and saline groups were prepared with the top 15 genes with the highest fold

change in enrichment cores of GSEA analyses using GraphPad Prism (version 10.2.2). GSEA Pathways were oxidative phosphorylation (OXPHOS) (GOBP), ribosome biogenesis (GOBP), unfolded protein response (UPR) (Hallmarks), sterol biosynthetic process (GOBP), lung fibrosis (Wikipathways), and fatty acid beta oxidation (GOBP). The PScan online software (version 1.6) [76] was used for the identification of over-represented transcription factor binding sites (TFBS) in the promoter associated with significant upregulated transcripts. Gene symbols were converted to Refseq mRNA accessions with db2db (bioDBnet) [77], promoter regions were from -950 to +50, and the descriptor was Jaspar 2020_NR.

SUPPLEMENTARY TABLES

Supplemental Table S1

List of antibodies used in the IHC and IF staining

Antibody	Source	Dilution	Unmasking	Incubation time (min)	
				Primary antibody	Secondary antibody
IKKβ	Novus Biological NB600-477	1/1000	H2	60	15
MOMA-2	Bio-Rad MCA519G	1/50	E1	30	15
MMP2	Abcam ab37150	1/100	H1	15	8
α-SMA	Invitrogen MA5-11547	1/50	H2	180	60
Gal3	Cederlane CL8942AP	1/100	H2	180	60

Supplemental Table S2

Sequences of the primers and UPL probes.

Gene	Accession number	UPL Probe	Oligo FWD	Oligo REV
Vcam1	NM_011693.3	34	tggtgaaatggaatctgaacc	cccagatggtggtttcctt
Icam1	NM_010493.2	81	cccacgctacctctgctc	gatggatacctgagcatcacc
Il1b	NM_008361.3	38	agttgacggaccccaaaag	agctggatgctctcatcagg
Ccl2	NM_011333.3	19	cttctgggcctgctgttc	gtggggcgttaactgcat
Mmp2	NM_008610.3	75	gcgcttttctcgaatccat	gggtatccatctccatgctc
Mmp9	NM_013599.2	53	cctgaaaacctccaacctca	ggtgtaaccatagcggtacaagt
Mmp14	NM_008608.3	42	gagaacttcgtgttgcctga	ctttgtgggtgaccctgact
Col1a2	NM_007743.2	80	ctggtgcacagggtgtga	ctcctgcttgacctggagtt
Col3a1	NM_009930.2	64	tggaccccaaggtcttcc	catctgatccagggtttcca
Timp1	NM_001044384.1	76	gcaaagagctttctcaaagacc	agggatagataaacagggaaacact
Lox	NM_010728.3	82	ctcctgggagtggcacag	cttgctttgtggccttcag
Loxl1	NM_010729.3	64	tatgcctgcacctctcacac	tgtccgcattgtatgtgtcat
Loxl2	NM_033325.2	69	gacctacaaccccaaagccta	gtgcccgtgcagttcatag
Loxl4	NM_001164311.1	15	gttgcacaactgccacaca	gggagtgcagtaatggcttc
P4ha2	NM_011031.2	78	ccgcctgctctctcttga	ccgttcaaagtaccgcagat
P4ha3	NM_177161.4	72	ctcgagagttccttgtctacagc	ttctcagccaagagcctttc
Tgfb1	NM_011577	72	tggagcaacatgtggaactc	gtcagcagccggttacca
Klf4	NM_010637.3	62	cgggaagggagaagacact	gagttcctcacgccaacg
Tagln	NM_011526.5	13	ccttccagtccacaaacgac	gtaggatggacccttgttgg
Myh11	NM_001161775	74	cagacctcatgcagctccaa	tctccaggtcagcttgcttg
Atg5	NM_053069	18	cactctctcgatggctgctt	ttcaatgtggacagcaagcta
Actb	NM_007393.3	56	aaggccaaccgtgaaaagat	gtggtacgaccagaggcatac
Hprt	NM_013556.2	95	tcctcctcagaccgctttt	cctggttcatcatcgctaatc

SUPPLEMENTARY FIGURES

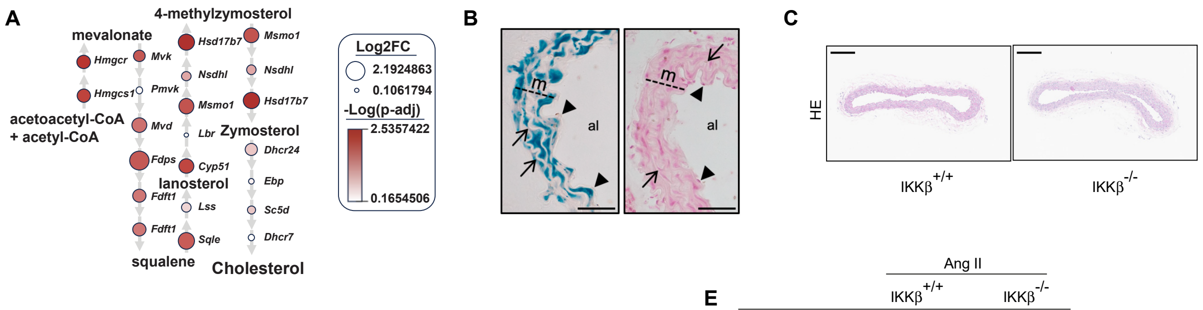

D

	IKKβ+/+	IKKβ-/-
Body weight, g	26.6 ± 0.6	27.6 ± 0.6
End point systolic blood pressure, mm Hg	165 ± 16	181 ± 15

E

	Ang II	
	IKKβ+/+	IKKβ-/-
Cholesterol, mg/dL	149	231
VLDL (7-16), mg/dL	44.3	48.3
LDL (17-32), mg/dL	64.8	97
HDL (33-50), mg/dL	35.3	66.3
Triglycerides, mg/dL	109 ± 90	230 ± 172

Supplemental Figure S1: Biochemical and pathophysiological characteristics of the IKKβ$^{+/+}$ and IKKβ$^{-/-}$ mice and ROSA26/ lacZ reporter mouse line.

A) Schematic of the enzymatic steps in cholesterol biosynthesis and the induction of associated transcripts by Ang II in wild-type mice after 24 hours of treatment. Bubble size is proportional to fold change (Log2), and colour indicates significance (-log10 p-value). For clarity, only selected metabolic intermediates are shown. B) Activation of SMA-CreER^{T2} upon tamoxifen injection is restricted to VSMC. Eight-week-old heterozygous SMA-CreER^{T2}ROSA26/lacZ$^{+/-}$ male mice were treated with either 1 mg tamoxifen (left panel) or vehicle (peanut oil; right panel) for 5 days. Isolated SRA regions were collected and subjected to X-Gal staining of 10 µm cryosections. Arrows: elastic fibres; arrowheads: endothelial cells; al: aortic lumen; m: media. Scale bars: 40 µm. C) Representative haematoxylin and eosin (HE) stains of SRA sections from IKKβ$^{+/+}$ (n = 2) and IKKβ$^{-/-}$ (n = 2) mice infused with saline for 28 days. Scale bars: 150 µm. D) Endpoint systolic blood pressure was measured during the last week of Ang II infusion at 1000 ng/kg/min for 28 days. Body weights were measured at the protocol endpoint. Data were quantified and presented as mean ± SEM. E) Total plasma cholesterol, very-low-density lipoprotein (VLDL) (FPLC fractions 7-16), low-density lipoprotein (LDL) (fractions 17-32), and high-density lipoprotein (HDL) (fractions 33-50) cholesterol were measured at the endpoint of Ang II infusion at 1000 ng/kg/min for 28 days. Data represent pooled plasma from 4 mice per group. Plasma triglyceride concentration was quantified in individual mice (n = 4/group) and presented as mean ± standard deviation.

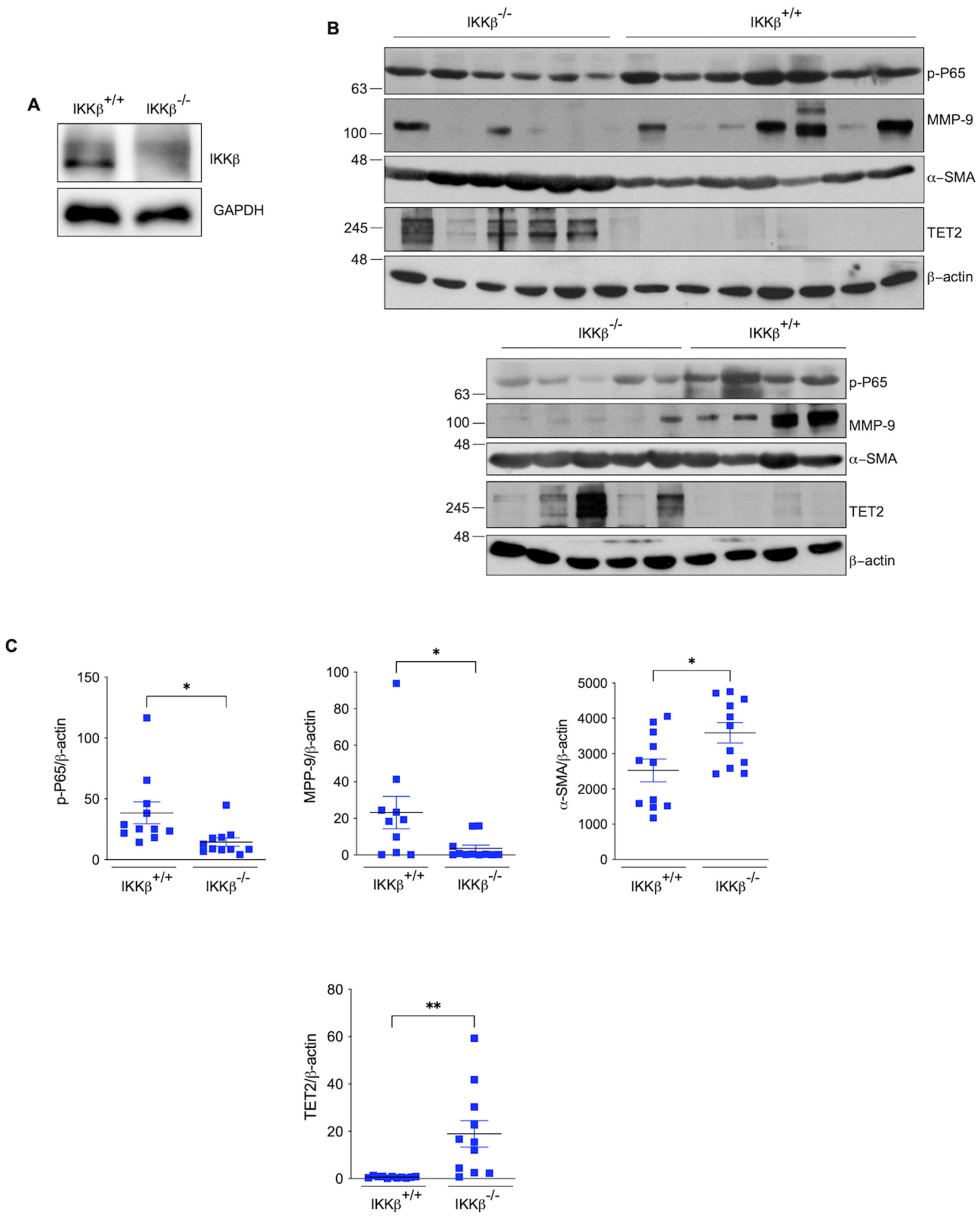
A
IKKβ+/+
IKKβ-/-
IKKβ
GAPDH
B
IKKβ-/-
IKKβ+/+
p-P65
MMP-9
α-SMA
TET2
β-actin
63
100
48
245
C
p-P65/β-actin
MPP-9/β-actin
α-SMA/β-actin
TET2/β-actin
*
**

Supplemental Figure S2: VSMC-specific IKKβ deletion in mice preserves their contractile phenotype. A) SRA regions from IKKβ $^{+/+}$ (n = 5) and IKKβ $^{-/-}$ (n = 5) mice were pooled, and proteins were extracted to validate IKKβ deletion. B) Proteins were extracted from SRA regions of IKKβ $^{+/+}$ (n = 11) and IKKβ $^{-/-}$ (n = 11) mice treated with Ang II for 28 days or that died suddenly from aortic rupture. Tissue extracts were subjected to immunoblotting with the indicated antibodies. For markers run on gels in which α-SMA was detected, the β-actin loading controls shown in the figure were obtained from separate membranes with identical samples, processed in parallel. This approach was necessary because the use of the α-SMA antibody altered the membrane, which could not be stripped properly. C) Data are presented as mean ± SEM. * $p < 0.05$, ** $p < 0.01$, **** $p < 0.0001$, 2-tailed Student's t test.

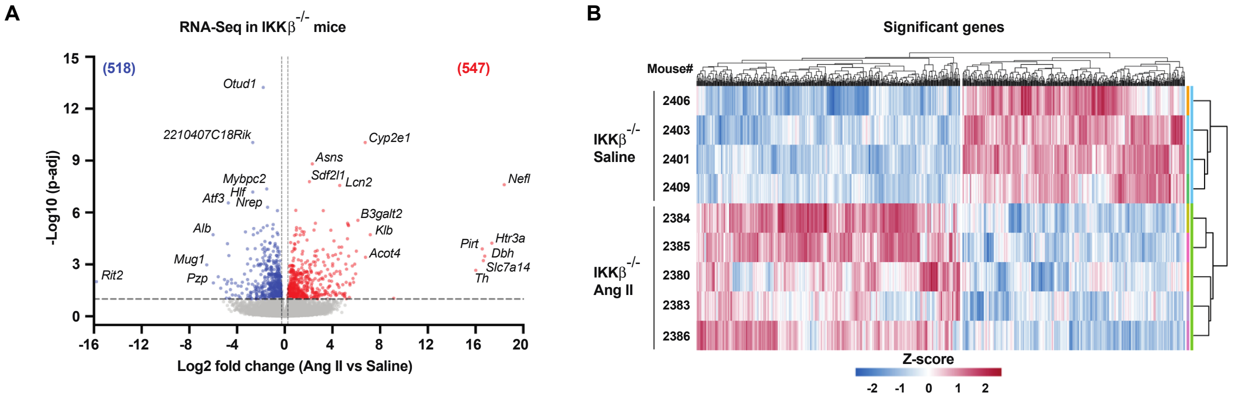
A
RNA-Seq in IKKβ-/- mice
(518)
(547)
Otud1
2210407C18Rik
Cyp2e1
Asns
Sdf2l1
Lcn2
Nefl
Mybpc2
Hlf
Atf3
Nrep
B3galt2
Klb
Alb
Pirt
Htr3a
Dbh
Acot4
Mug1
Slc7a14
Pzp
Th
Rit2
-Log10 (p-adj)
Log2 fold change (Ang II vs Saline)
B
Significant genes
Mouse#
IKKβ-/- Saline
2406
2403
2401
2409
IKKβ-/- Ang II
2384
2385
2380
2383
2386
Z-score
-2 -1 0 1 2

Supplemental Figure S3: IKKβ$^{-/-}$ mice still respond to Ang II. A) Volcano plot of differentially expressed transcripts (DET) in the SRA of IKKβ$^{-/-}$ mice after 24 hours of Ang II treatment. Each point shows the mean fold change and the associated p-adjusted value for a single transcript, comparing Ang II (n=5 mice) with saline (n=4 mice). Coloured numbers indicate the number of significantly induced (red) and reduced (blue) transcripts at the given threshold (dotted lines), as described in Materials and Methods. B) Heatmap of significant DET. Expression fold changes have been transformed to z-scores, and clustering has been performed on rows (mice) and columns (transcripts).

A

Groups	Deaths	Deaths due to AAA rupture	Deaths due to TAA rupture	Deaths due to both AAA and TAA rupture
IKKβ+/+	15/21	6/15 (40%)	2/15 (13%)	7/15 (47%)
IKKβ-/-	7/30	3/7 (43%)	1/7 (14%)	3/7 (43%)

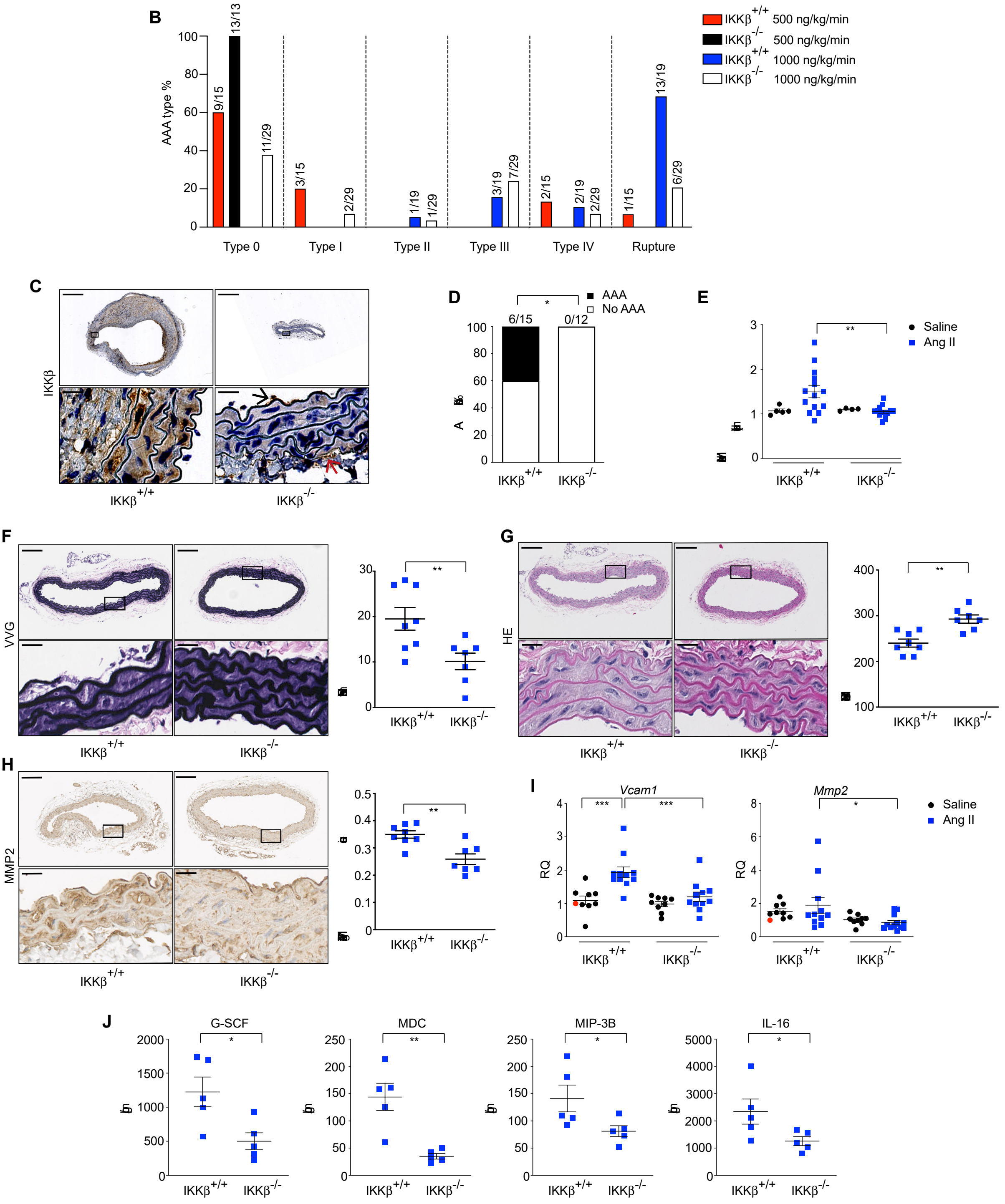

Supplemental Figure S4: IKKβ expression in vascular SMC is necessary for AAA development and fatal rupture. A) Cause of death in both groups infused with Ang II at 1000 ng/kg/min for 28 days. TAA: thoracic aortic aneurysm. B) AAA classification in both groups infused with Ang II at 500 ng/kg/min or 1000 ng/kg/min for 28 days. C) Representative histochemistry of conditional IKKβ deletion in VSMC of SRA sections from IKKβ$^{+/+}$ (n = 2) and IKKβ$^{-/-}$ (n = 2) mice infused with 500 ng/kg/min of Ang II for 28 days. Black arrow and red arrow indicate intimal and adventitial staining, respectively, demonstrating specific IKKβ knockout in the media. Scale bars: 600 µm; magnification scale bars: 37.5 µm. D) AAA incidence in mice from both groups infused with Ang II at the protocol endpoint. * $p < 0.05$, Fisher's exact test. E) Maximal abdominal aortic diameter in mice from both groups infused with saline or Ang II at the protocol endpoint. Data were quantified and presented as mean ± SEM. ** $p < 0.01$, 2-way ANOVA with Tukey's multiple-comparison test. F) Representative Verhoeff–Van Gieson (VVG) stains of SRA sections from IKKβ$^{+/+}$ (n = 8) and IKKβ$^{-/-}$ (n = 7) mice infused with Ang II, with elastin breaks counted. Scale bars: 150 µm; magnification scale bars: 37.5 µm. Data were quantified and presented as mean ± SEM. ** $p < 0.01$, 2-tailed Student's t test. G) Representative haematoxylin and eosin (HE) stains of SRA sections from both IKKβ$^{+/+}$ (n = 8) and IKKβ$^{-/-}$ (n = 7) mice infused with Ang II, with medial VSMC count. Scale bars: 150 µm; magnification scale bars: 37.5 µm. Data were quantified and presented as mean ± SEM. ** $p < 0.01$, 2-tailed Student's t test. H) Representative MMP2 immunohistochemistry of SRA sections from IKKβ$^{+/+}$ (n = 8) and IKKβ$^{-/-}$ (n = 7) mice infused with Ang II, with scoring. Scale bars: 150 µm; magnification scale bars: 37.5 µm. Data were quantified and presented as mean ± SEM. ** $p < 0.01$, 2-tailed Student's t test. I) qPCR analysis of *Vcam1* and *Mmp2* mRNA expression in adSRA lysates from IKKβ$^{+/+}$ (n = 9) and IKKβ$^{-/-}$ (n = 9) mice infused with saline, and from IKKβ$^{+/+}$ (n = 11) and IKKβ$^{-/-}$ (n = 13) mice infused with Ang II at the protocol endpoint. Red circles represent the reference. Data were quantified and presented as mean ± SEM. * $p < 0.05$, *** $p < 0.001$, 2-way ANOVA with Tukey's multiple-comparison test. RQ: relative quantification. J) Levels of circulating neutrophil/macrophage chemokines from both groups infused

with Ang II ($n = 5$). Data were quantified and presented as mean ± SEM. * $p < 0.05$, ** $p < 0.01$, 2-tailed Student's *t*-test.
